# Frequent Monitoring of C-Peptide Levels in Newly Diagnosed Type 1 Subjects Using Dried Blood Spots Collected at Home

**DOI:** 10.1210/jc.2018-00500

**Published:** 2018-05-31

**Authors:** Ruben H Willemsen, Keith Burling, Peter Barker, Fran Ackland, Renuka P Dias, Julie Edge, Anne Smith, John Todd, Boryana Lopez, Adrian P Mander, Catherine Guy, David B Dunger

**Affiliations:** 1 University of Cambridge, Department of Paediatrics, Cambridge University Hospitals NHS Foundation Trust, Cambridge Biomedical Campus, Cambridge, United Kingdom; 2 Department of Paediatric Diabetes and Endocrinology, Royal London Hospital, Barts Health NHS Trust, London, United Kingdom; 3 NIHR Cambridge Biomedical Research Centre, Core Biochemistry Assay Laboratory, Cambridge University Hospitals NHS Foundation Trust, Cambridge Biomedical Campus, Cambridge, United Kingdom; 4 Paediatrics, Northampton General Hospital NHS Trust, Northampton, United Kingdom; 5 Department of Paediatric Endocrinology and Diabetes, Birmingham Children’s Hospital NHS Foundation Trust, Steelhouse Lane, Birmingham, United Kingdom; 6 Institutes of Metabolism and Systems Research, Vincent Drive, University of Birmingham, Birmingham, United Kingdom; 7 Centre for Endocrinology, Diabetes and Metabolism, Vincent Drive, Birmingham Health Partners, Birmingham, United Kingdom; 8 Paediatric Endocrinology, Oxford Radcliffe Hospitals NHS Trust, Headington, Oxford, United Kingdom; 9 JDRF/Wellcome Trust Diabetes and Inflammation Laboratory, Wellcome Trust Centre for Human Genetics, Nuffield Department of Medicine, NIHR Oxford Biomedical Research Centre, University of Oxford, Oxford, United Kingdom; 10 University of Cambridge MRC Biostatistics Unit Hub for Trials Methodology Research, Cambridge, United Kingdom; 11 Wellcome Trust MRC Institute of Metabolic Science, Cambridge, United Kingdom

## Abstract

**Objective:**

To evaluate an approach to measure *β*-cell function by frequent testing of C-peptide concentrations in dried blood spots (DBSs).

**Patients:**

Thirty-two children, aged 7 to 17 years, with a recent diagnosis of type 1 diabetes.

**Design:**

Mixed-meal tolerance test (MMTT) within 6 and again at 12 months after diagnosis, with paired venous and DBS C-peptide sampling at 0 and 90 minutes. Weekly DBS C-peptide before and after standardized breakfasts collected at home.

**Results:**

DBS and plasma C-peptide levels (n = 115) correlated strongly (*r* = 0·91; *P* < 0.001). The Bland-Altman plot indicated good agreement. The median number of home-collected DBS cards per participant was 24 over a median of 6.9 months. Repeated DBS C-peptide levels varied considerably within and between subjects. Adjustment for corresponding home glucose measurements reduced the variance, permitting accurate description of changes over time. The correlation of the C-peptide slope over time (assessed by repeated home DBS) vs area under the curve during the two MMTTs was *r* = 0.73 (*P* < 0.001). Mixed models showed that a 1-month increase in diabetes duration was associated with 17-pmol/L decline in fasting DBS C-peptide, whereas increases of 1 mmol/L in glucose, 1 year older age at diagnosis, and 100 pmol/L higher baseline plasma C-peptide were associated with 18, 17, and 61 pmol/L higher fasting DBS C-peptide levels, respectively. In addition, glucose responsiveness decreased with longer diabetes duration.

**Conclusion:**

Our approach permitted frequent assessment of C-peptide, making it feasible to monitor *β*-cell function at home. Evaluation of changes in the slope of C-peptide through this method may permit short-term evaluation of promising interventions.

## Teaser

Frequent C-peptide measurements in subjects with newly diagnosed type 1 diabetes through the use of dried blood spots at home provide a convenient and reliable method for monitoring *β*-cell function.

Our knowledge of the natural history of type 1 diabetes (T1D) has changed in the last few years with the increasing awareness that a period of dysglycemia and reduced *β*-cell glucose sensitivity may occur between the first appearance of autoantibodies and the development of disease (1–3). Even when T1D has developed, the rate of immune-mediated destruction of *β*-cells may vary, with some subjects becoming C-peptide depleted soon after presentation and others maintaining some degree of *β*-cell function for many years after diagnosis (4–8). Thus, our ability to monitor changes in *β*-cell function over the short and long term may be critical to our understanding of disease pathophysiology and the evaluation of potentially useful interventions to prevent development and progression of the disease (9).

C-peptide is an excellent marker of residual *β*-cell activity, and even random or fasting levels may be of clinical significance because they have been associated in longitudinal studies with HbA1c, the risk of microvascular complications, and the incidence of hypoglycemia (8, 10–12). Little is known about the day-to-day variation in random or fasting C-peptide levels in people who developed T1D or who are in the prediabetic period. It has been assumed that such data are likely to be highly variable and that more formal assessments of residual *β*-cell function are needed for the evaluation of therapeutic interventions. The mixed-meal tolerance test (MMTT) and glucagon stimulation tests have been shown to provide a good measure of *β*-cell function (13), and a US Food and Drug Administration draft guidance suggested that the repeated MMTT might be the best standardized measure of drug efficacy in phase 2 and 3 clinical trials of potentially useful interventions (14). A basic protocol based on repeated MMTTs over 1 to 3 years has remained the benchmark for all subsequent clinical studies in prediabetes and newly diagnosed T1D.

However, the MMTT is labor intensive and requires admission to a clinical research facility for several hours. It is therefore probably not the best test to obtain repeated measures of C-peptide over short periods of time. There is increasing interest in determining how environmental or short-term drug exposures affect *β*-cell function. To develop methods that might be suitable for this purpose we have examined the value of fasting and single postprandial measures of C-peptide collected from dried blood spots (DBSs) in the home setting. We report longitudinal changes in fasting and postprandial C-peptide in young people recently diagnosed with T1D and contrast the information gained about change in C-peptide over time with that obtained by more formal interval MMTTs.

## Subjects and Methods

### Subjects

Thirty-two subjects with T1D, aged 7 to 17 years, with ≥1 autoantibody positive test in their local laboratory, necessitating insulin treatment, were recruited within 1 to 24 weeks after diagnosis. Exclusion criteria were type 2, monogenic, or secondary diabetes, use of immunosuppressive agents or oral steroids in the last 2 months, pregnancy, and celiac disease. Four participants withdrew before and two withdrew at the second MMTT (one unable to cannulate, one unable to perform MMTT because of hyperglycemia and not wanting to be rescheduled).

### Study design

**Figure 1.**
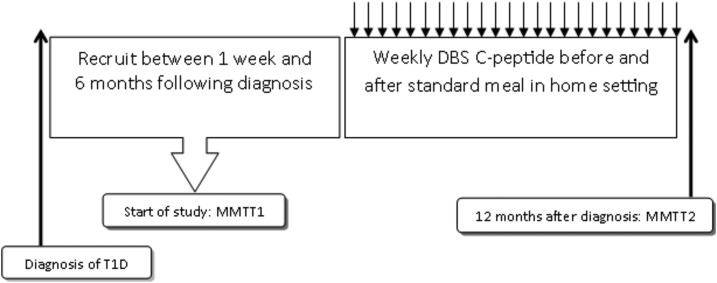
Study design.

The patients underwent two MMTTs (Fig. 1): the first one at recruitment (any time between 1 and 24 weeks from diagnosis) (visit 1) and the second 12 months from diagnosis (visit 2) (Fig. 1). In between, they were asked to collect weekly DBSs at home before (fasting) and 90 minutes after a standardized breakfast, with paired recordings of capillary glucose on their own glucometer. Written and verbal instructions for DBS collection were provided at visit 1. Participants were instructed to let the DBS card dry for 24 hours without using heat or direct sunlight and to mail the cards to the laboratory via prepaid envelopes. Participants were provided with a list of standardized breakfasts containing 35, 45, and 50 g of carbohydrates for 5- to 6-, 7- to 12-, and 13- to 18-year-olds, respectively, and were asked to pick a breakfast that they could eat on a weekly basis.

### MMTT

The MMTT was performed after an overnight fast (from midnight), with no food or drink other than water. Long-acting insulin and basal rates (for insulin pump users) were continued as normal. The use of rapid-acting insulin was acceptable up to ≤2 hours before the MMTT. The MMTT was performed only if the participant’s glucose level was 4 to 11.1 mmol/L. Participants ingested 6 mL/kg of Boost meal solution (maximum 360 mL; Nestlé Healthcare Nutrition), within 10 minutes. Blood samples for C-peptide and glucose were collected 10 minutes before the meal (−10 minutes), at time of ingestion (0 minutes), and at 15, 30, 60, 90, and 120 minutes.

Two capillary DBS samples were taken during the MMTT: between −10 and 0 minutes and at 90 minutes for comparison with plasma samples.

### Other assessments

At each MMTT, height and weight were measured and body mass index (BMI) was calculated. Age- and sex-appropriate standard deviation scores were calculated for height, weight, and BMI (15, 16). HbA1c, insulin regimen, and total daily dose of insulin per kilogram [(mean insulin requirements over the last 3 days)/weight] were recorded at both MMTT visits.

### Laboratory methods

Plasma C-peptide samples taken during the MMTT were assayed in singleton on a DiaSorin Liaison® XL automated immunoassay analyzer with a one-step chemiluminescence immunoassay. Between-batch imprecision for the assay is 6.0% at 561 pmol/L (n = 181), 4.3% at 2473 pmol/L (n = 170), and 5.4% at 5400 pmol/L (n = 166).

DBS cards were stored at −80°C until analysis. DBS C-peptide was analyzed in duplicate with a modification of the Meso Scale Discovery two-step electrochemical immunoassay (Meso Scale Diagnostics custom human C-peptide kit). Two 3.2-mm diameter spots were punched for each standard, quality control, and sample and eluted into 80 µL Diluent 13 (Meso Scale Diagnostics) by shaking for 1 hour at 4°C. A portion of this elution buffer (60 µL) was transferred to the analytical plate, and the standard two-step immunoassay was performed.

Whole blood C-peptide DBS standards were created with washed red cells diluted to produce a packed cell volume of ~0.45 in 5% BSA 1× PBS containing a variety of dilutions of the C-peptide international reference reagent. Aliquots of each of these prepared whole blood standards were centrifuged and the plasma assayed in triplicate with the DiaSorin Liaison XL assay. The average result for each standard was used as the assigned value for that DBS standard. The remainder of the whole blood standards were spotted out on several cards, allowed to dry, and stored at −80°C. A card was defrosted, punched as necessary, and, if there was sufficient sample left for further runs, refrozen at −80°C. This process was repeated for each run of the assay. Each card could last four or five runs. Quality control data analyzed over a 7-month period showed no noticeable trend in results, indicating good stability over time (Supplemental Fig. 1). Between-batch imprecision for the DBS assay was 8.7% at 451 pmol/L (n = 115), 10.0% at 495 pmol/L (n = 115), and 11.3% at 878 pmol/L (n = 113). The lower limit of detection (LLD) was 50 pmol/L. Values recorded as lower than LLD were assigned 25 pmol/L.

Glucose levels taken at the MMTT were analyzed via an adaption of the hexokinase-glucose-6-phosphate dehydrogenase method (17). For the home glucose measurements, patients used various glucometers.

### Ethics

The National Research Ethics Committee East of England–Cambridge South approved the study. All patients ≥16 years old and all parents gave informed consent, and children <16 years gave assent to the study procedures.

### Statistical analyses

Data are expressed as mean (SD) or median [interquartile range (IQR)], depending on normality of data distribution. Changes in parameters between visit 1 and 2 were evaluated by a paired Student *t* test or Wilcoxson paired rank test.

Intraclass correlation was used to compare the paired plasma and DBS samples collected during the MMTT. A power calculation showed that, based on a two-sided test, a sample size of 30, allowing for a dropout rate of 20%, *α* = 5%, provided >90% power to detect a correlation coefficient of ≥0.6 between plasma and DBS C-peptide levels. In addition, a Bland-Altman plot (the differences between MMTT DBS and plasma C-peptide plotted against the mean of those measurements) was produced to compare the plasma and DBS measurements. Because the differences between DBS and plasma C-peptide were not normally distributed, data were log transformed before the mean and the difference were calculated.

For the correlation analysis and Bland-Altman plot, all available paired samples were used (n = 115; three missing DBS samples). For the analyses of postprandial DBS C-peptide and DBS C-peptide increment, only the home recordings were used, because the samples collected during the MMTT visits were collected after a different stimulus and without insulin administration.

To compare changes in *β*-cell function across the two methods [area under the curve (AUC) C-peptide during MMTT vs postprandial home DBS C-peptide], we estimated slopes by regressing each estimate of *β*-cell function on duration of diabetes. The estimated slopes were then divided by their respective SDs to standardize the measurements in scale. This produces a measurement akin to a *z* score, because the measurements have been standardized in scale but not centered at the mean. Bland-Altman plots were produced to compare the two methods. Correlation was calculated via Pearson correlation.

To investigate the effects of diabetes duration, glucose levels, baseline plasma C-peptide, age at diagnosis, and sex on DBS C-peptide, mixed-effects regression models were used with random intercepts to account for the repeat measurements per person. In these models diabetes duration, glucose levels, baseline plasma C-peptide, and age at diagnosis were entered as covariates and sex as a factor. To examine whether there was a change in glucose responsiveness with longer duration of diabetes, we tested whether there was an interaction between diabetes duration and glucose levels. To examine whether there was a more pronounced decline in C-peptide with longer diabetes duration in younger children, we tested whether there was an interaction between age at diagnosis and diabetes duration.

Statistical analysis was performed in SPSS version 23 (IBM SPSS Statistics) and R version 1.0.136 (R Project for Statistical Computing).

## Results

### Baseline data

Table 1 shows parameters of all participants at visits 1 and 2. HbA1c deteriorated slightly over time. The insulin dosage did not change significantly. Fasting and 90-minute glucose levels increased significantly. As expected, all estimates of *β*-cell function (DBS C-peptide, AUC, and peak plasma C-peptide derived from the MMTT) decreased significantly over time.

**Table 1.** Baseline Data at MMTT Visits 1 and 2

	Visit 1	Visit 2	*P*
N	32	28	—
Ethnicity	Asian 3	Asian 3	—
	Mixed 3	Mixed 3	
	White 26	White 22	
Age at diagnosis, y	12.0 (2.9)	11.9 (3.1)	—
Age at visit, y	12.4 (2.9)	12.9 (3.1)	<0.001
Sex, male/female	12/20	9/19	—
Duration of diabetes, d*^a^*	154 (65)	365 (9)	<0.001
HbA1c, mmol/mol	51 (12)	59 (20)	<0.05
Height SDS	0.19 (1.0)	0.15 (1.0)	NS
BMI SDS	0.62 (1.0)	0.64 (1.2)	NS
Insulin regimen			
Basal bolus	27	18	—
Pump	5	9	—
≤3 Injections	0	1	—
Insulin dose, U/kg/d	0.57 (0.23)	0.64 (0.28)	NS
Fasting glucose, mmol/L*^a^*	6.5 (2.3)	8.1 (2.8)	<0.01
Fasting DBS C-peptide, pmol/L*^a^*	379 (396)	245 (240)	<0.05
90-min glucose, mmol/L*^a^*	15.4 (4.0)	19.0 (3.9)	<0.001
90-min DBS C-peptide, pmol/L*^a^*	948 (727)	493 (641)	<0.001
Mean AUC C-peptide, pmol/L*^a^*	0.59 (1.76)	0.34 (0.45)	<0.001
Peak C-peptide in MMTT, pmol/L*^a^*	794 (582)	422 (660)	<0.001

Abbreviations: NS, not significant; SDS, SD score.

^a^ Values reported as median (IQR); all other values reported as mean (SD).

### Comparison of the plasma and DBS C-peptide method

**Figure 2.**
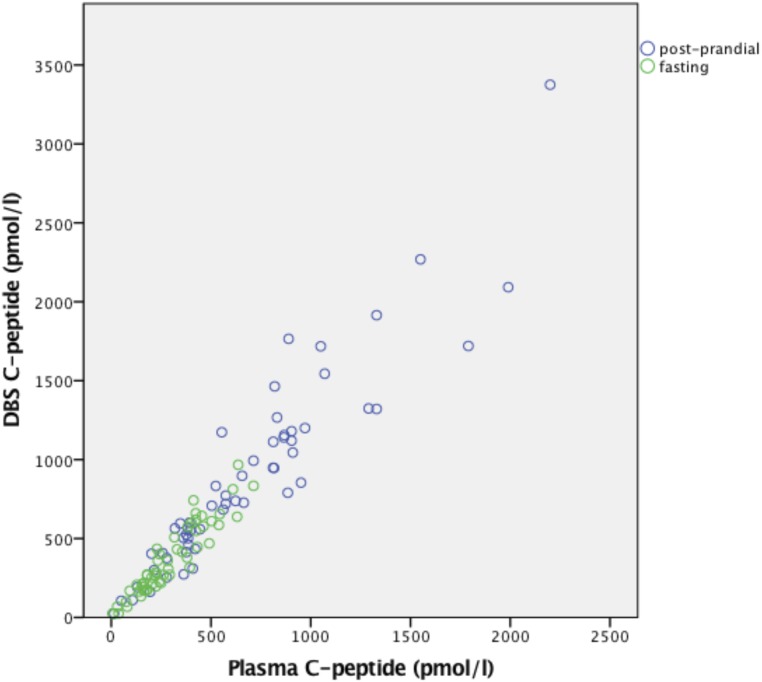
Correlation between plasma and DBS MMTT C-peptide levels. Fasting and postprandial values indicated by green and blue circles, respectively. N = 115; *r* = 0.91; *P* < 0.001.

**Figure 3.**
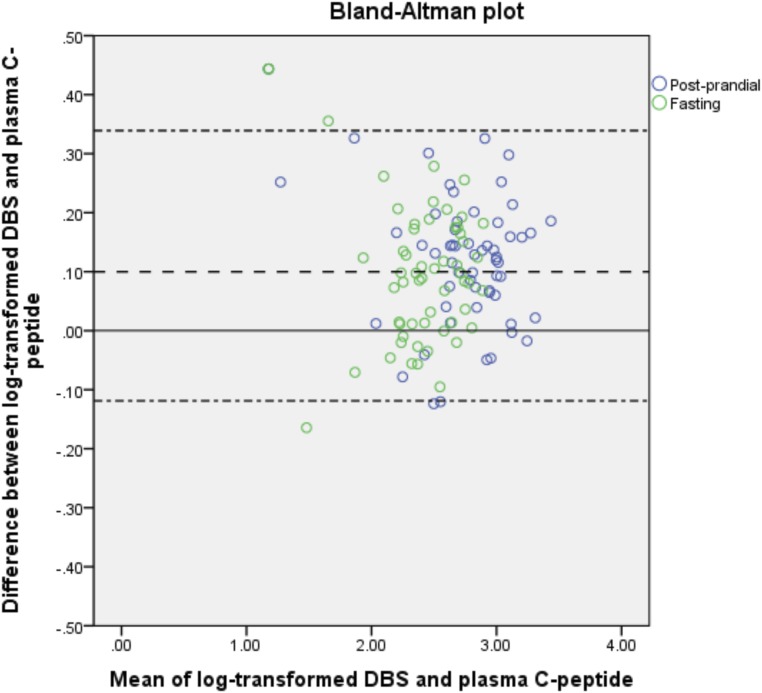
Bland-Altman plot for the comparison of the DBS and plasma C-peptide assay, performed on paired plasma and DBS samples during MMTT.

DBS and plasma C-peptide levels correlated strongly (n = 115 paired samples; *r* = 0.91; *P* < 0.001; Fig. 2). The Bland-Altman plot is shown in Fig. 3. The DBS method slightly overestimated C-peptide levels with a mean (SD) difference of 1.27 (1.31) times the plasma values (*P* < 0.001). The 95% limits of agreement were 0.76 to 2.18.

### DBS C-peptide measurements

For those who completed the study up to the second MMTT, the median number of home DBS samples per participant was 24 (minimum 8 and maximum 40), collected over a median duration of 6.9 months (IQR 1.8). The median (IQR) interval between collection and receipt of the DBS cards in the laboratory was 3 days. All but two participants had detectable C-peptide levels throughout the study. Supplemental Fig. 2 shows the course of fasting and postprandial DBS C-peptide measurements over time for each subject.

### Comparison of slopes defined by the different methods to estimate the change in *β*-cell function over time

**Figure 4.**
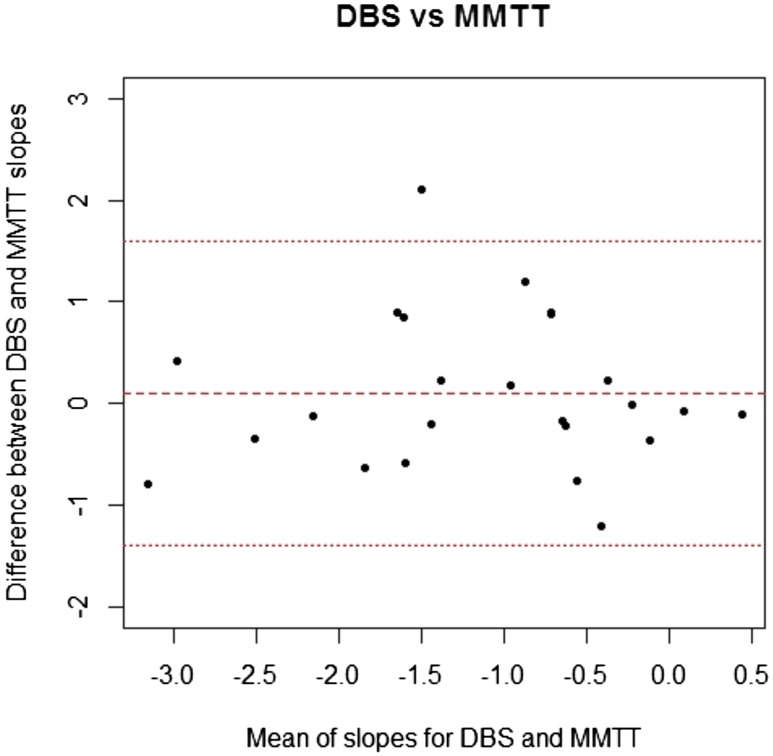
Bland-Altman plot for the comparison of the slopes in *β*-cell function across the two different methods (AUC C-peptide during MMTT vs postprandial home DBS C-peptide).

We compared the change in *β*-cell function over time as estimated by the AUC plasma C-peptide during the MMTT with postprandial home DBS C-peptide, respectively, by constructing a Bland-Altman plot where the slopes derived from the two methods were compared (Fig. 4). The mean (SD) difference in slopes was 0.10 (0.75) for DBS vs MMTT (not significantly different from zero). The correlation of the slopes between the two methods (DBS vs MMTT) was *r* = 0.73, *P* < 0.001.

### Covariates of fasting DBS C-peptide

In a series of models we evaluated covariates of fasting DBS C-peptide (Table 2). Diabetes duration had a significant negative effect on fasting DBS C-peptide. Fasting glucose, age at diagnosis, and baseline fasting C-peptide had significant positive effects on subsequent DBS C-peptide levels (models 1 and 2). Sex did not have a significant effect (data not shown).

**Table 2.** Covariates of Fasting and Postprandial DBS C-Peptide

Fasting DBS C-Peptide								
	Model 1		Model 2		Model 3		Model 4	
	*β*	*P*	*β*	*P*	*β*	*P*	*β*	*P*
Diabetes duration, d	−0.57	<0.001	−0.57	<0.001	−1.2	<0.01	−1.8	<0.001
Fasting glucose, mmol/L	17.6	<0.001	17.9	<0.001	45.1	<0.001	43.5	<0.001
Age at diagnosis, y	25.7	0.025	17.2	0.037	16.8	0.044	7.4	0.433
Baseline plasma C-peptide, pmol/L			0.61	<0.001	0.60	<0.001	0.60	<0.001
Diabetes duration × fasting glucose*^a^*					−0.10	<0.001	−0.10	<0.001
Diabetes duration × diabetes duration*^a^*					0.003	<0.001	0.003	<0.001
Diabetes duration × age at diagnosis*^a^*							0.04	0.051
Akaike information criterion	8941		8920		8903		8901	

Postprandial DBS C-Peptide								
	Model 1		Model 2		Model 3		Model 4	
	*β*	*P*	*β*	*P*	*β*	*P*	*β*	*P*
Diabetes duration, d	−1.4	<0.001	−1.4	<0.001	−1.9	0.016	−1.7	0.081
Postprandial glucose, mmol/L	14.8	<0.001	14.7	<0.001	48.6	<0.001	49.4	<0.001
Age at diagnosis, y	53.5	0.031	34.0	0.076	35.5	0.067	38.9	0.069
Baseline plasma C-peptide, pmol/L			1.2	<0.001	1.2	<0.001	1.2	<0.001
Diabetes duration × postprandial glucose*^a^*					−0.13	<0.001	−0.13	<0.001
Diabetes duration × diabetes duration*^a^*					0.004	0.023	0.004	0.023
Diabetes duration × age at diagnosis*^a^*							−0.01	0.716
Akaike information criterion	8860		8845		8828		8830	

^a^ Interaction terms: To investigate the effects of covariates on DBS C-peptide, we used a series of mixed-effects regression models with random intercepts to account for the repeat measurements per person. The interaction terms in these models indicate whether the coefficient of a covariate changes with longer diabetes duration. For example, a negative coefficient for the interaction term “diabetes duration × glucose” indicates that the (positive) effect of glucose on DBS C-peptide decreases with longer duration of diabetes. To examine whether there was a change in glucose responsiveness with longer duration of diabetes, we tested whether there was an interaction between diabetes duration and glucose levels. To examine whether there was a more pronounced decline in C-peptide with longer diabetes duration in younger children, we tested whether there was an interaction between age at diagnosis and diabetes duration.

Consequently, we investigated whether glucose responsiveness changed over time by adding an interaction term (model 3), and this step further improved the model, indicating that the C-peptide response to a certain glucose level gets weaker with longer diabetes duration (Supplemental Fig. 3a). Diabetes duration as a quadratic term also improved the model, indicating that the decline in DBS C-peptide was not linear over time (model 3). Finally, we tested whether age at diagnosis affected the slope of decline of C-peptide by adding an interaction term (model 4), and this effect reached borderline significance, indicating that older children had a less steep decline in C-peptide over time. In the model without interactions (model 2), 1-month longer diabetes duration was associated with a 17-pmol/L decline in fasting DBS C-peptide, whereas increases of 1 mmol/L in glucose, 1 year older age at diagnosis, and 100 pmol/L higher baseline plasma C-peptide were associated with 18-, 17-, and 61-pmol/L higher fasting DBS C-peptide levels, respectively.

### Covariates of postprandial DBS C-peptide

Diabetes duration had a significant negative effect on postprandial DBS C-peptide levels, whereas fasting glucose and age at diagnosis had a significant positive effect on DBS C-peptide levels (Table 2; model 1). Adding baseline fasting C-peptide to the model weakened the association with age at diagnosis (model 2), probably because of confounding. Sex did not have a significant effect (data not shown). As in the fasting DBS C-peptide analyses, glucose responsiveness decreased with longer diabetes duration (Supplemental Fig. 3b), and the decline in DBS postprandial C-peptide over time appeared to be nonlinear (model 3). Age at diagnosis did not affect the slope of decline of postprandial C-peptide over time (model 4). In the model without interactions (model 2), a 1-month longer diabetes duration was associated with a 42-pmol/L decline in postprandial DBS C-peptide, whereas increases of 1 mmol/L in glucose, 1 year older age at diagnosis, and 100 pmol/L higher baseline plasma C-peptide were associated with 15-, 34-, and 120-pmol/L higher postprandial DBS C-peptide levels, respectively.

### Covariates of DBS C-peptide increment after breakfast

Diabetes duration and fasting DBS C-peptide had a significant negative effect on the C-peptide response to breakfast, and the glucose increment after breakfast had a significant positive effect on the C-peptide response (Table 3; model 1). Sex and age at diagnosis were not significant (data not shown). By adding an interaction term, we showed that with longer duration of diabetes there was a lower C-peptide response to a unit glucose increment (model 2; Supplemental Fig. 3c). In the model without interactions (model 1), a 1-month longer duration of diabetes was associated with a 30-pmol/L decline in the DBS C-peptide increment, whereas an increase of 1 mmol/L in the delta glucose was associated with a 16-pmol/L increase in the DBS C-peptide increment, corrected for fasting DBS C-peptide levels.

**Table 3.** Covariates of DBS C-Peptide Increment After Breakfast

	Model 1		Model 2	
	*β*	*P*	*β*	*P*
Diabetes duration, d	−1.0	<0.001	−0.69	<0.001
Delta glucose, mmol/L	16.3	<0.001	48.3	<0.001
Fasting DBS C-peptide, pmol/L	−0.44	<0.001	−0.45	<0.001
Diabetes duration × delta glucose*^a^*			−0.12	<0.001
Akaike information criterion	8709		8694	

^a^ Interaction terms: To investigate the effects of covariates on DBS C-peptide, we used a series of mixed-effects regression models with random intercepts to account for the repeat measurements per person. The interaction terms in these models indicate whether the coefficient of a covariate changes with longer diabetes duration. For example, a negative coefficient for the interaction term “diabetes duration × glucose” indicates that the (positive) effect of glucose on DBS C-peptide decreases with longer duration of diabetes. To examine whether there was a change in glucose responsiveness with longer duration of diabetes, we tested whether there was an interaction between diabetes duration and glucose levels. To examine whether there was a more pronounced decline in C-peptide with longer diabetes duration in younger children, we tested whether there was an interaction between age at diagnosis and diabetes duration.

Tables 2 and 3 show results for fasting DBS C-peptide and postprandial DBS C-peptide (Table 2) and the DBS C-peptide increment (Table 3), respectively.

## Discussion

In this study we developed an approach to measure C-peptide in DBS in children and adolescents with recently diagnosed T1D and compared it with the MMTT. The DBS method showed a strong correlation with the plasma C-peptide method, and the Bland-Altman plot indicated good performance of the DBS assay. A median number of 24 received DBS cards per participant indicated that the method was feasible in the home setting. In addition, this method permitted frequent assessment of C-peptide, which allows accounting for biological variation and facilitates short-term evaluations of possible interventions.

C-peptide has been measured in DBS before and shown to remain stable on filter paper cards for 6 months (18). However, the LLD of the previous assay (18) was 440 pmol/L, compared with 50 pmol/L in the current study. This greater sensitivity is important because residual concentrations of ≥200 pmol/L in patients with T1D protect against diabetes complications (10, 12, 19).

The frequent assessment of C-peptide levels in our participants highlighted the biological variation in C-peptide levels in children and adolescents with T1D. A significant proportion of this variation could be explained by the concurrent glucose levels, with higher glucose levels associated with higher C-peptide levels. In studies that use the MMTT to evaluate *β*-cell function, the glucose excursion during an MMTT is normally not taken into account, but our data suggest that this excursion affects measured C-peptide levels. Future studies evaluating potential biomarkers of an individual patient’s change in *β*-cell function over time could adjust for concurrent glucose levels.

By investigating which covariates had an effect on DBS C-peptide levels, we demonstrated, as expected, that longer diabetes duration was associated with lower DBS C-peptide levels. The decline in C-peptide over time was not linear but curved, which has been described before in a study that used MMTTs, but in that study the slope of decline in *β*-cell function changed only after a 12-month diabetes duration (5). Concurrently measured glucose levels had a positive effect on C-peptide levels. In addition, we demonstrated that this positive effect of glucose on C-peptide levels decreases with longer diabetes duration. This relationship was apparent in all three models, in which fasting DBS C-peptide, postprandial DBS C-peptide, or the DBS C-peptide increment was used as the dependent variable. This finding indicates that the ability of the pancreas to secrete more insulin in response to higher glucose levels, also called glucose responsiveness or glucose sensitivity (20), decreases with longer duration of diabetes. A decrease in this glucose sensitivity was found previously to be an early and strong predictor of progression to T1D in at-risk family members (20). The DBS method has not been evaluated yet in at-risk individuals, but the fact that changes in glucose responsiveness could be picked up is encouraging.

As expected, greater age at diagnosis was associated with higher fasting and postprandial C-peptide levels. We also demonstrated that younger children had a steeper decline in fasting C-peptide levels, in line with the results of a large study including 3929 patients from seven European registries that demonstrated a more rapid decline in fasting plasma C-peptide in patients with an earlier onset of T1D (21). Ludvigsson *et al.* (7) reported a greater difference between random, nonfasting C-peptide at diagnosis and at 1 year from diagnosis for those with a younger age at diagnosis, suggesting that younger children have a more rapid decline in *β*-cell function. The decline in C-peptide seemed particularly pronounced in children <5 years old at diagnosis (7). Greenbaum *et al.* (5) also reported a slower decline in *β*-cell function in subjects >21 years old compared with younger patients but a similar rate of decline in patients aged 7 to 21 years.

A limitation of our study is the fact that we did not ask the participants to withhold insulin in the home setting, and doing so may have influenced the amplitude of the prandial C-peptide response. We anticipated that omitting insulin at home would be too disruptive and might negatively affect adherence to the study. In retrospect this concern was unfounded, as indicated by the high number of collected DBS cards. Previous research has shown that the peak C-peptide in an MMTT with concurrent insulin administration, though lower, was still highly correlated to the peak in an MMTT without insulin (22). Another limitation is that the participants did not have the exact same stimulus for C-peptide secretion at home, because their breakfasts may have differed in fat and protein content, although we ensured age-banded, comparable amounts of carbohydrates in the breakfast across all participants, and the weekly breakfast for each participant was the same.

Our study highlights the heterogeneous nature of changes in *β*-cell function in children and adolescents with T1D. Some participants showed a dramatic decline in *β*-cell function within the first year from diagnosis, whereas others did not. There were only two participants whose *β*-cell function became undetectable during the study. The identification of biomarkers that can predict the course of *β*-cell function within the first year from diagnosis will be of major importance, because it would indicate which participants are likely to benefit most from interventions. In a previous study, sCD25, an established marker of immune activation and inflammation, was found to be elevated in patients with T1D as compared with controls and was also negatively associated with C-peptide levels (23). This marker and other markers of immune activity such as C-reactive protein could also be measured from DBS. Simultaneous measurement of metabolic and immune status could be particularly valuable in future attempts to stratify patients and in the frequent monitoring of the effects of potential therapeutics.

There is increasing interest in interventions to preserve or even restore residual *β*-cell function in patients with T1D (9). Some drugs approved for treatment of type 2 diabetes have been investigated in pediatric T1D, such as thiazolidinediones (24) and glucagon-like peptide 1 receptor agonists (25), and a growing number of immunotherapy studies (26–31) are being conducted. Some T1D trials have reported some, if temporary, beneficial effects on *β*-cell function (26, 27, 29, 31), although this effect led to reductions in insulin requirements in only a minority of patients (29, 31). Other trials have reported beneficial effects in adults but not in children (28) and with the opposite trend for a promising approach, inhibition of the T cell receptor CD3 (32). T1D is a heterogeneous disease, and it is likely that a more tailored, stratified approach is needed to improve treatment success. Such adaptive and dynamic treatment approaches are on their way (33), and a straightforward, less labor-intensive method permitting more frequent assessment of *β*-cell function may aid in the evaluation of interventions. Frequent assessment of C-peptide via DBS enables us to calculate a slope reflecting the change in *β*-cell function over time. The characterization of an individual patient’s slope after diagnosis may help in selecting participants who are likely to benefit most from intervention trials, and analyzing changes in this slope may be useful in the short-term evaluation of promising interventions. Finally, the DBS method may be useful in monitoring *β*-cell function in autoantibody-positive at-risk patients, a feature of T1D research receiving increasing attention (34).

## Supplementary Material

Supplemental Figures

## Abbreviations:

AUC: area under the curve
BMI: body mass index
DBS: dried blood spot
IQR: interquartile range
LLD: lower limit of detection
MMTT: mixed-meal tolerance test
T1D: type 1 diabetes
