# Supplementary material for: Frequent Monitoring of C-Peptide Levels in Newly Diagnosed Type 1 Subjects Using Dried Blood Spots Collected at Home
Source: J Clin Endocrinol Metab. 2018 May 31;103(9):3350–8. doi: 10.1210/jc.2018-00500 (PMC6126892; doi:10.1210/jc.2018-00500)

Supplementary Figure 1. Quality control data assessing the between batch variability at different standards of DBS C-peptide (A: 8·7% at 451 pmol/L (n=115); B: 10·0% at 495 pmol/L (n=115), and C: 11·3% at 878 pmol/L (n=113)). Dashed lines indicate 95% confidence intervals.


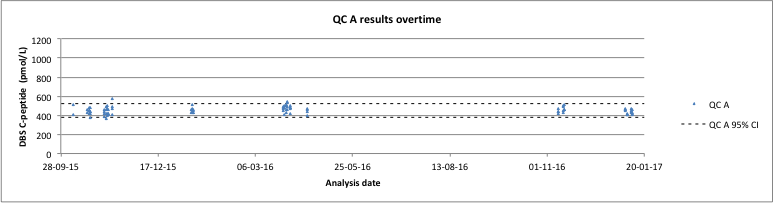

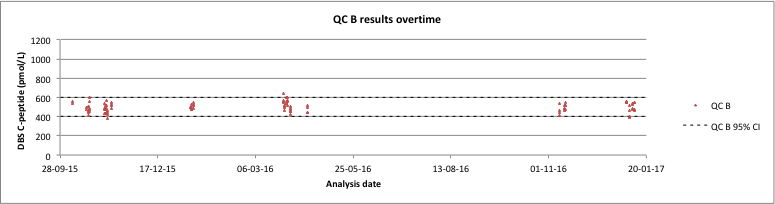

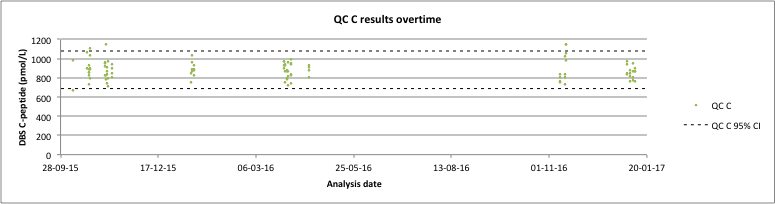


**Supplementary Figure 2. Individual participants’ course of fasting and post-prandial DBS C-peptide vs. diabetes duration**


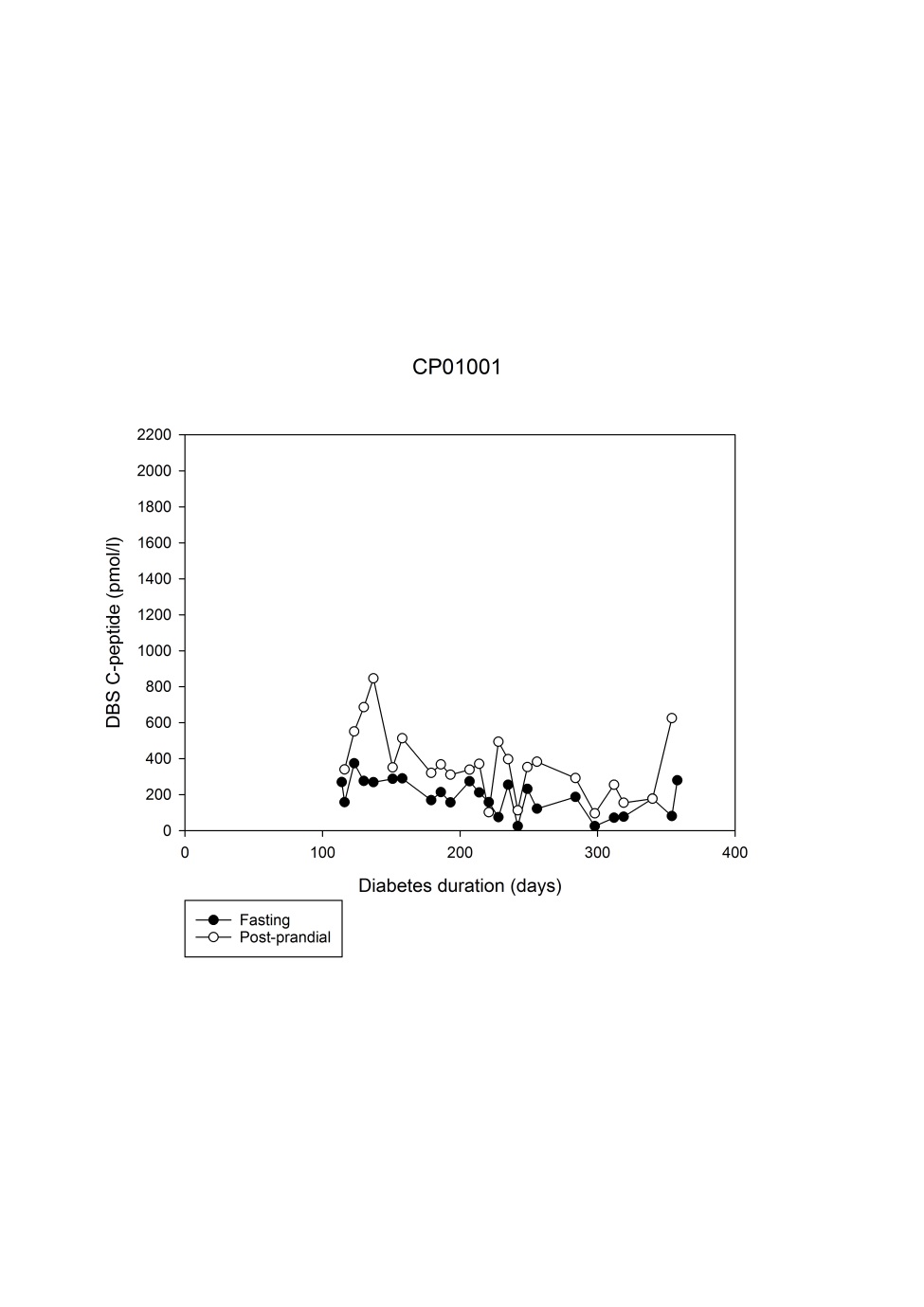

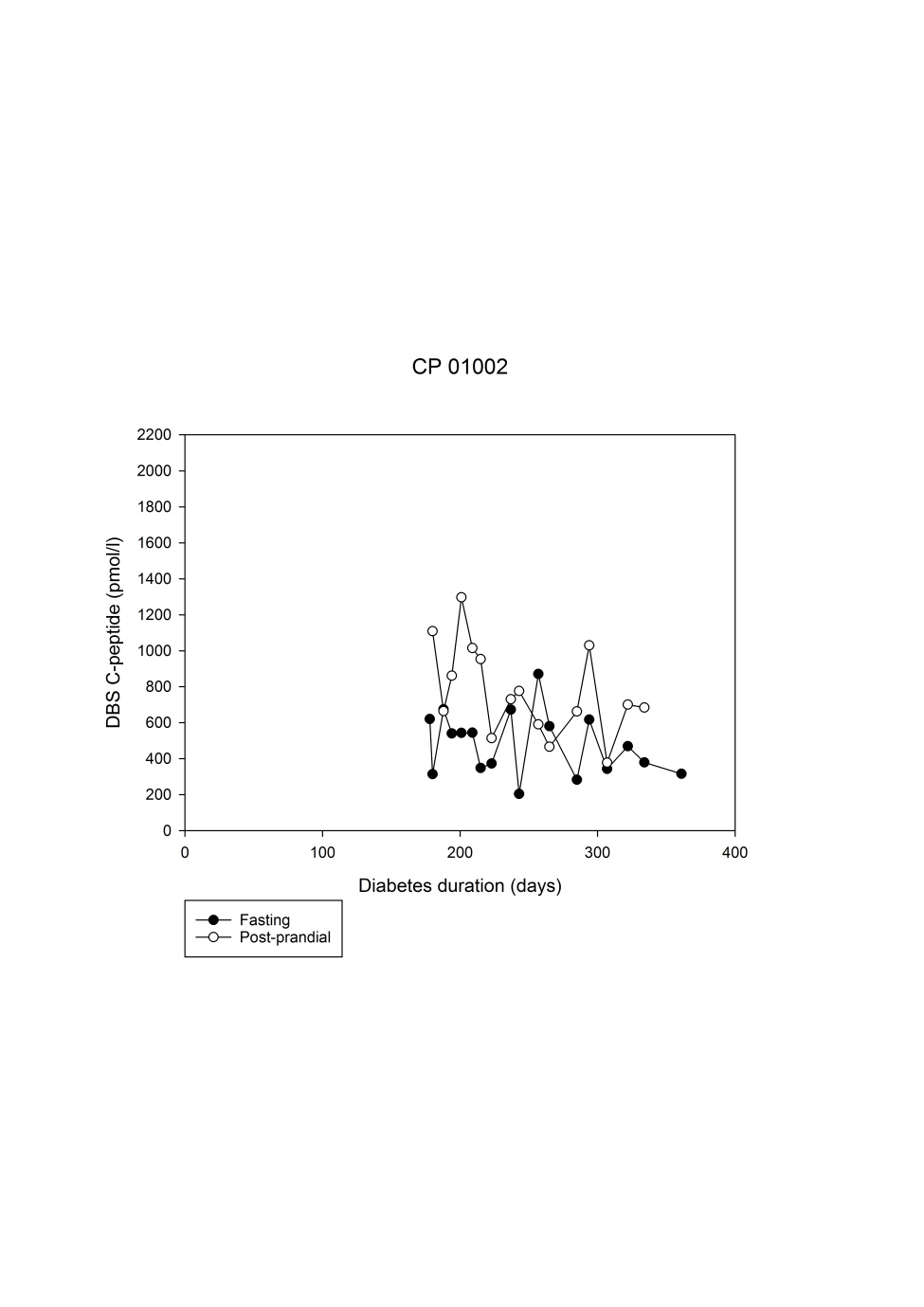

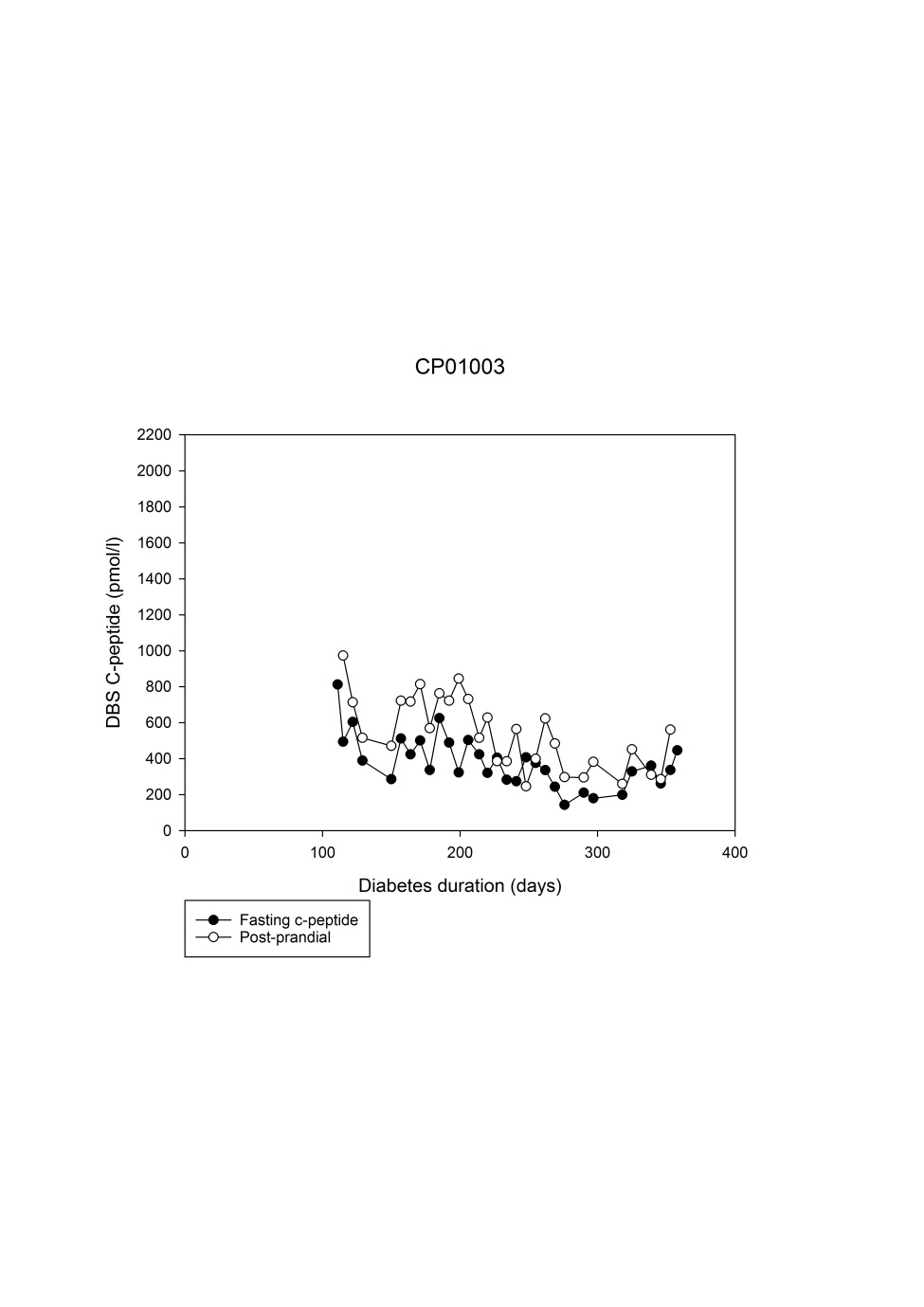

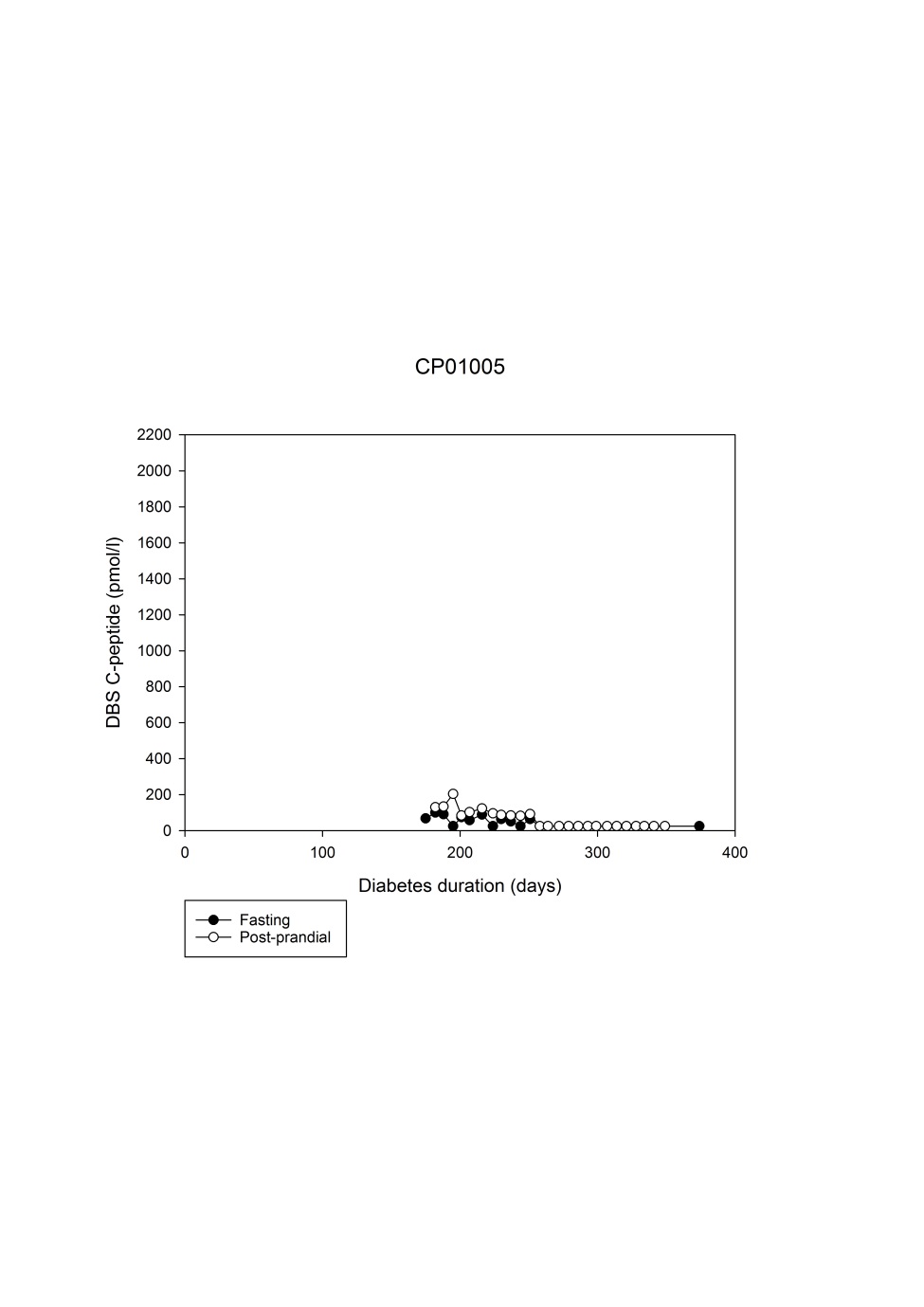


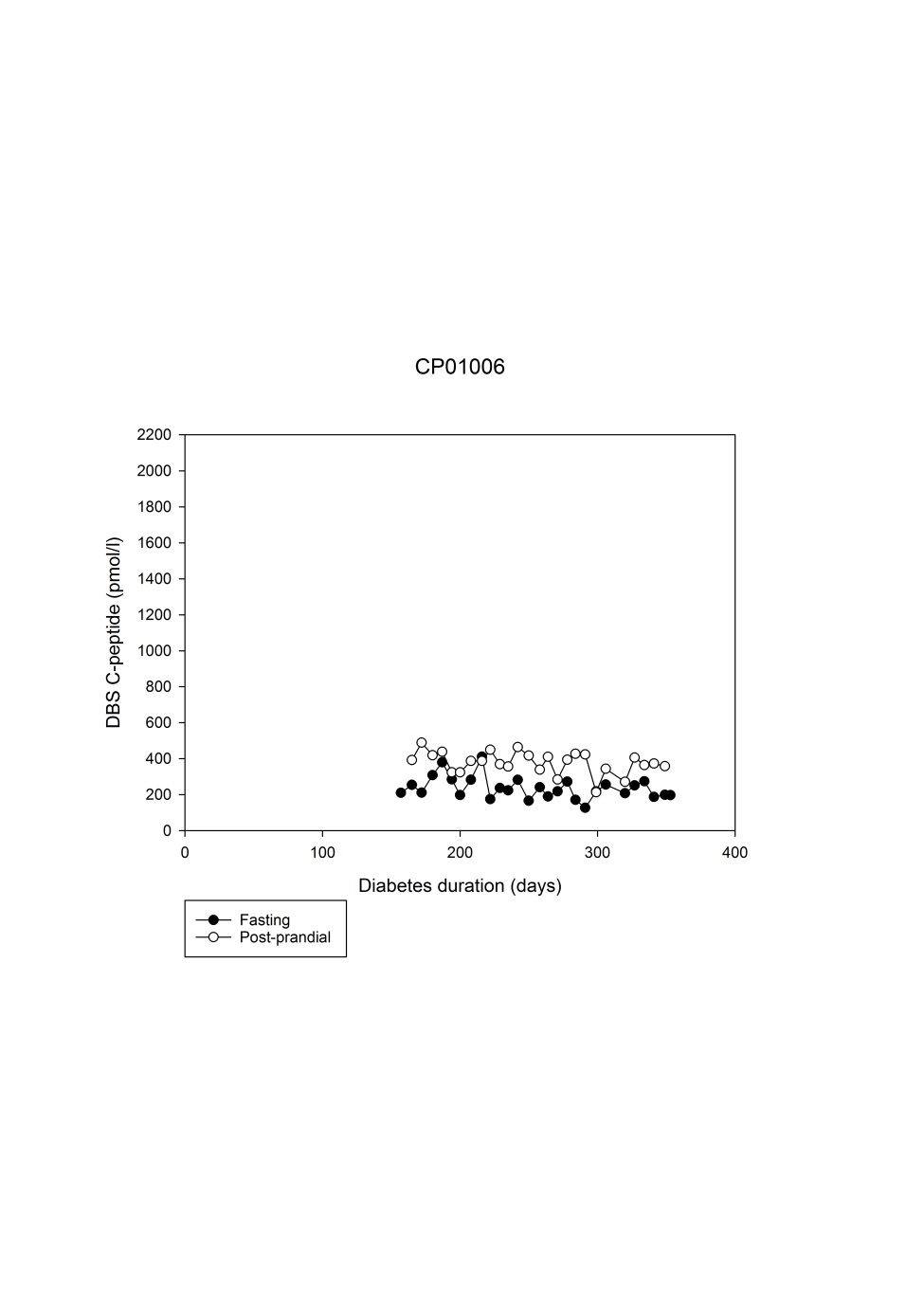

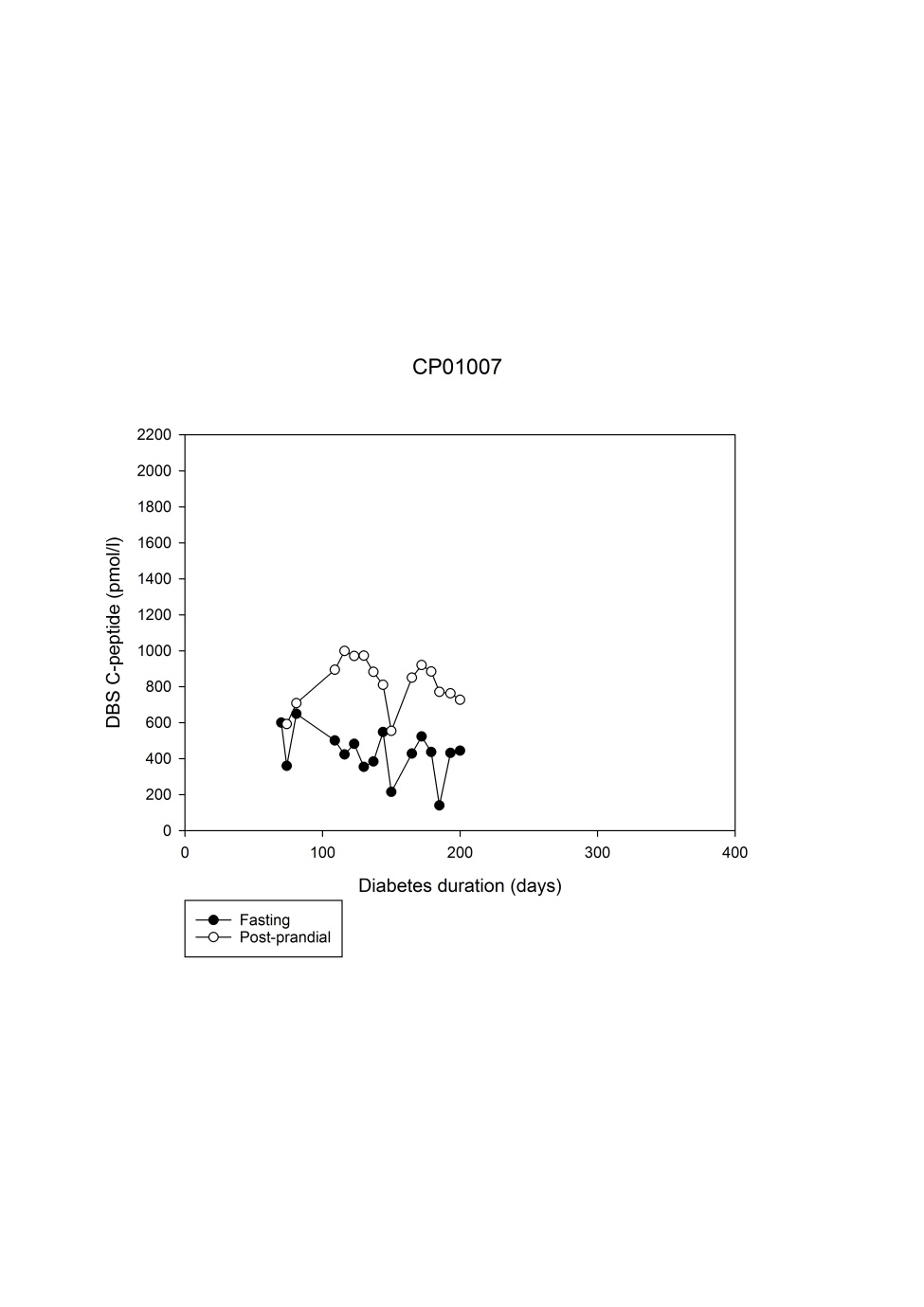

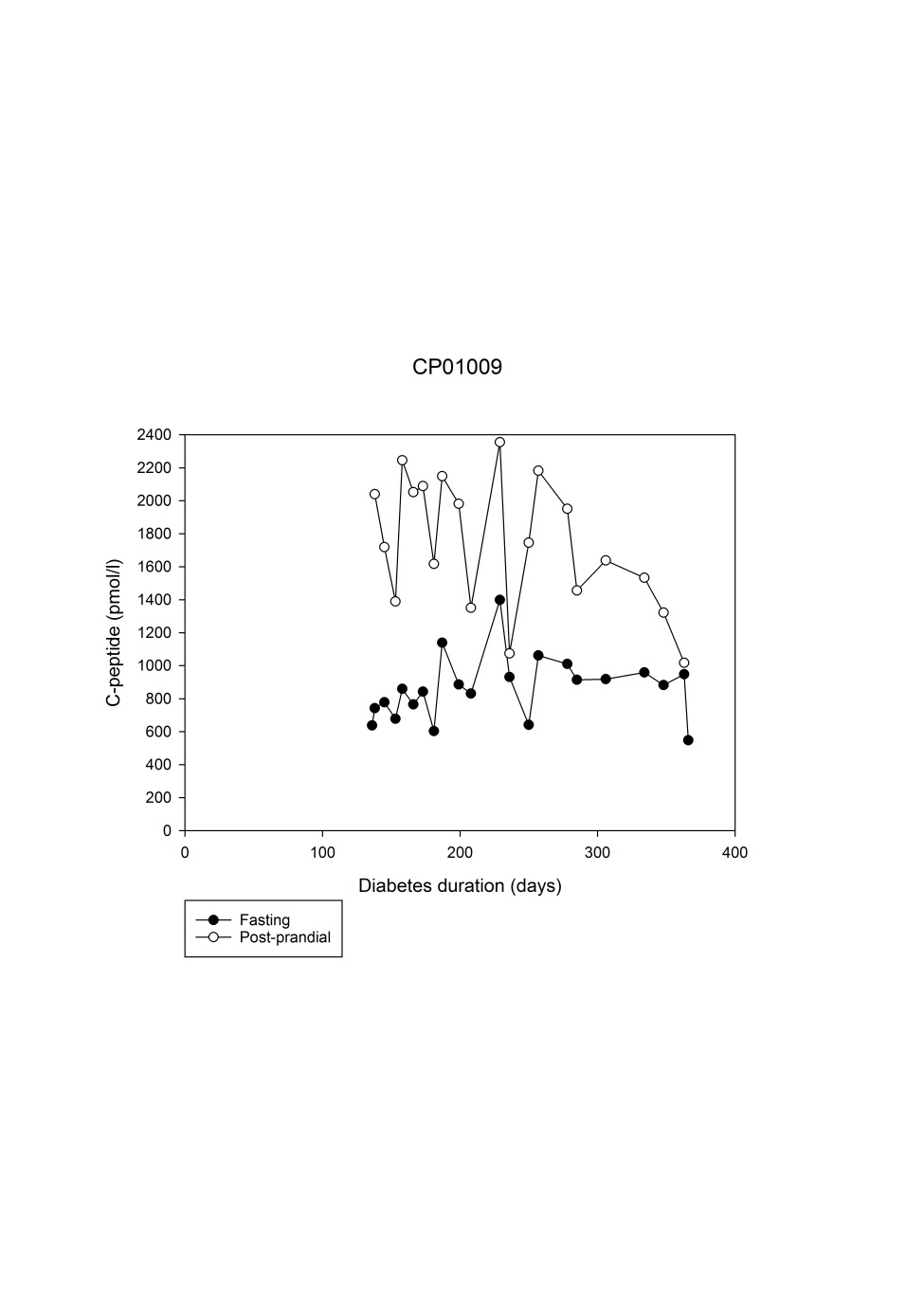

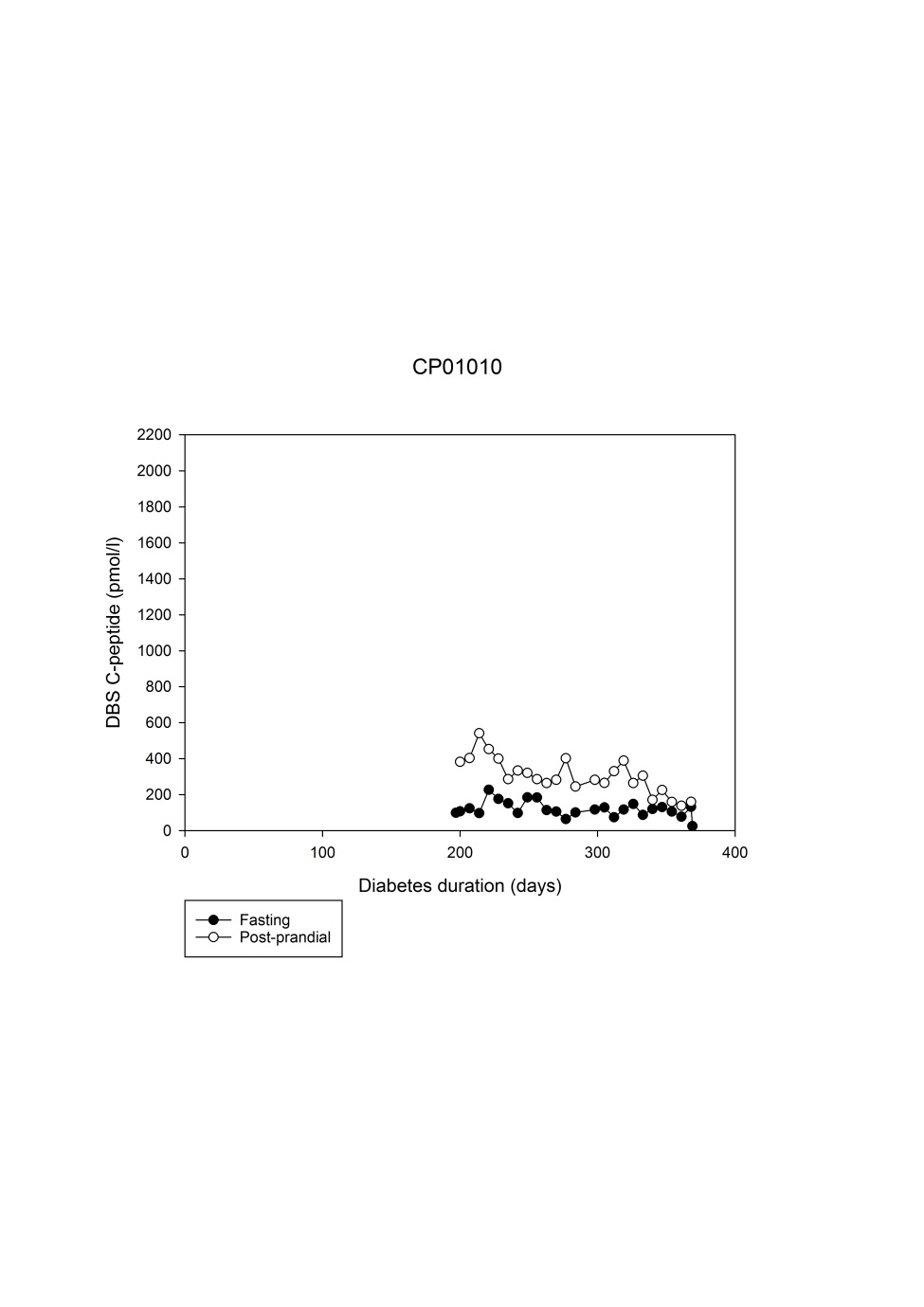

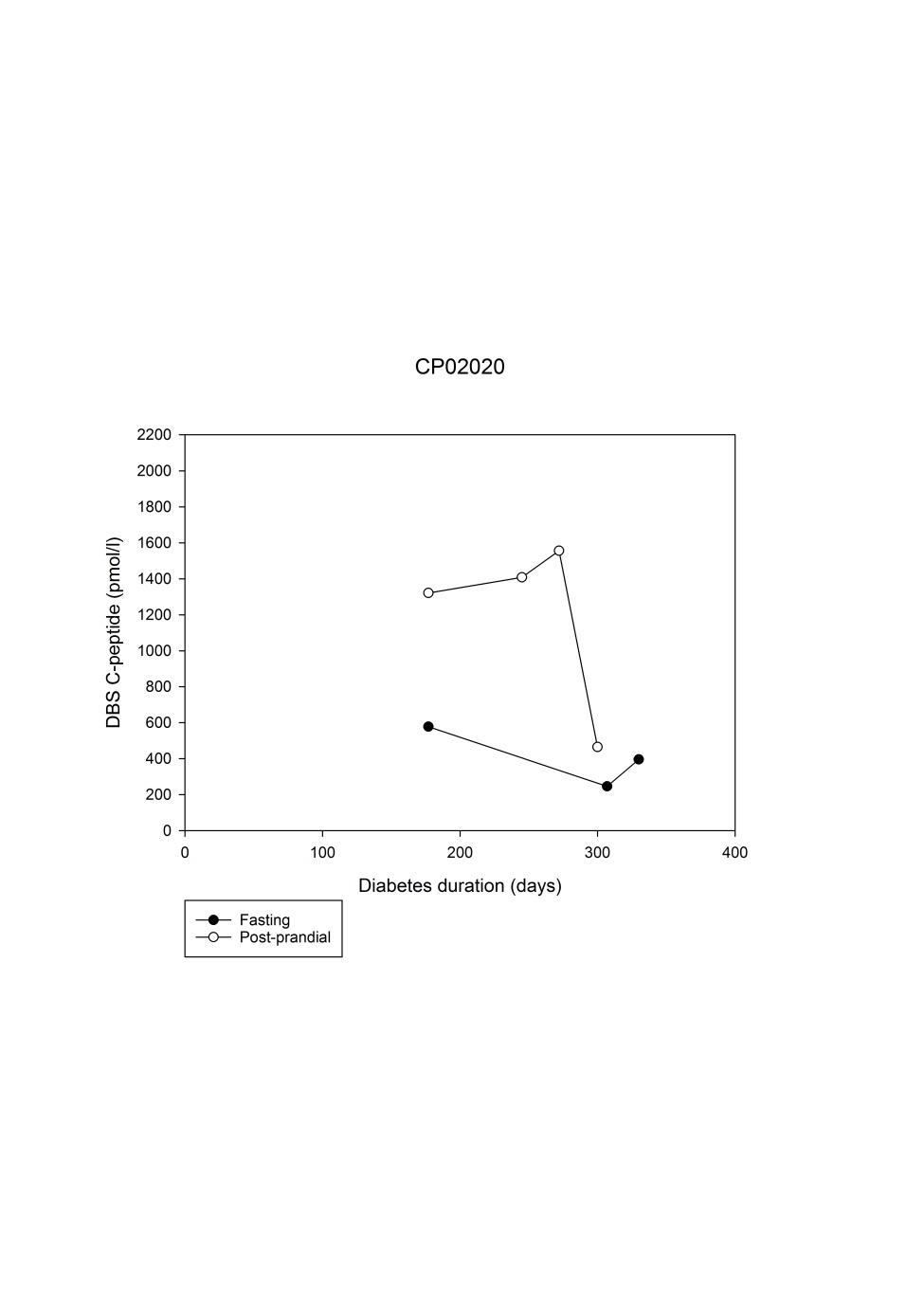

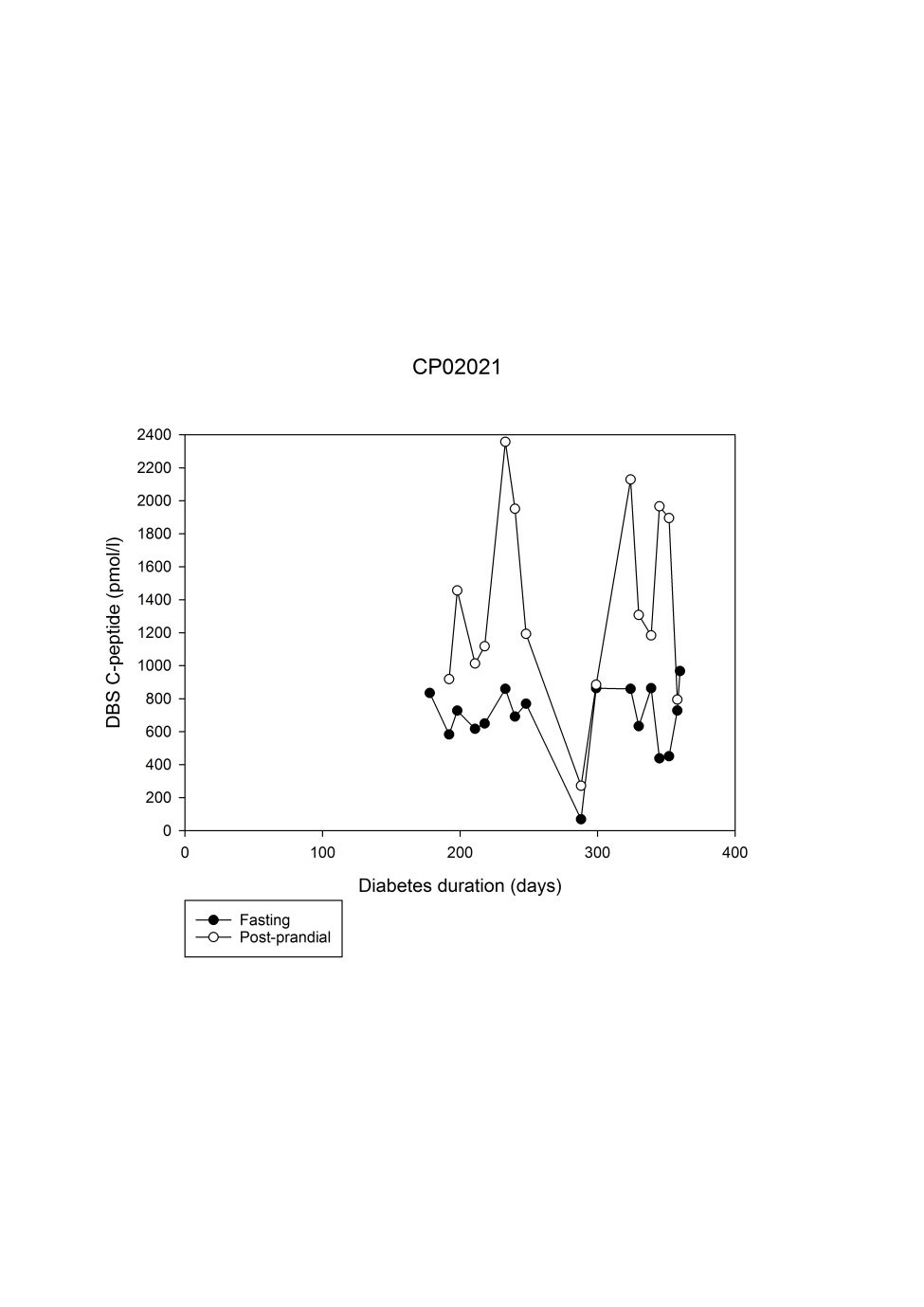

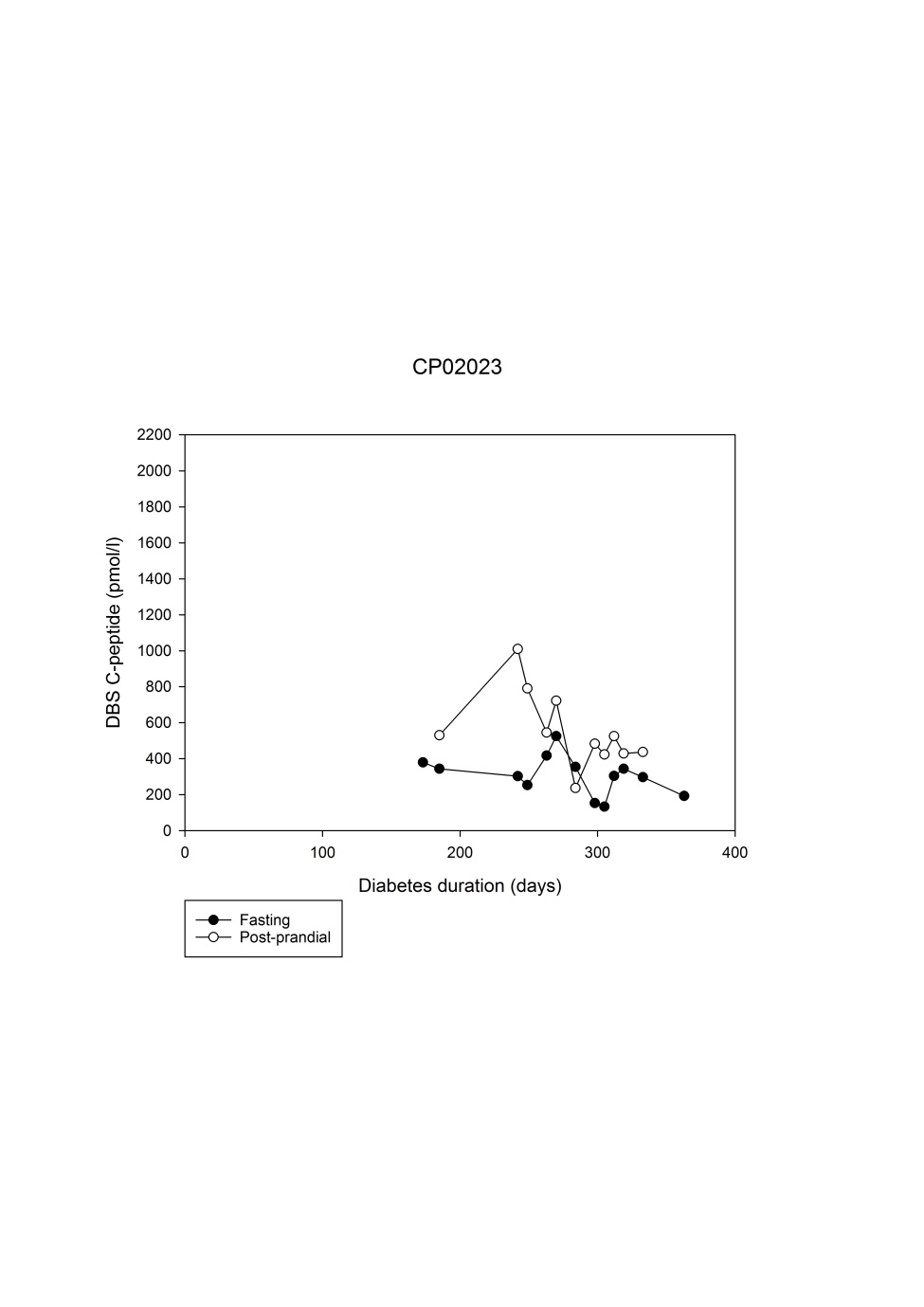

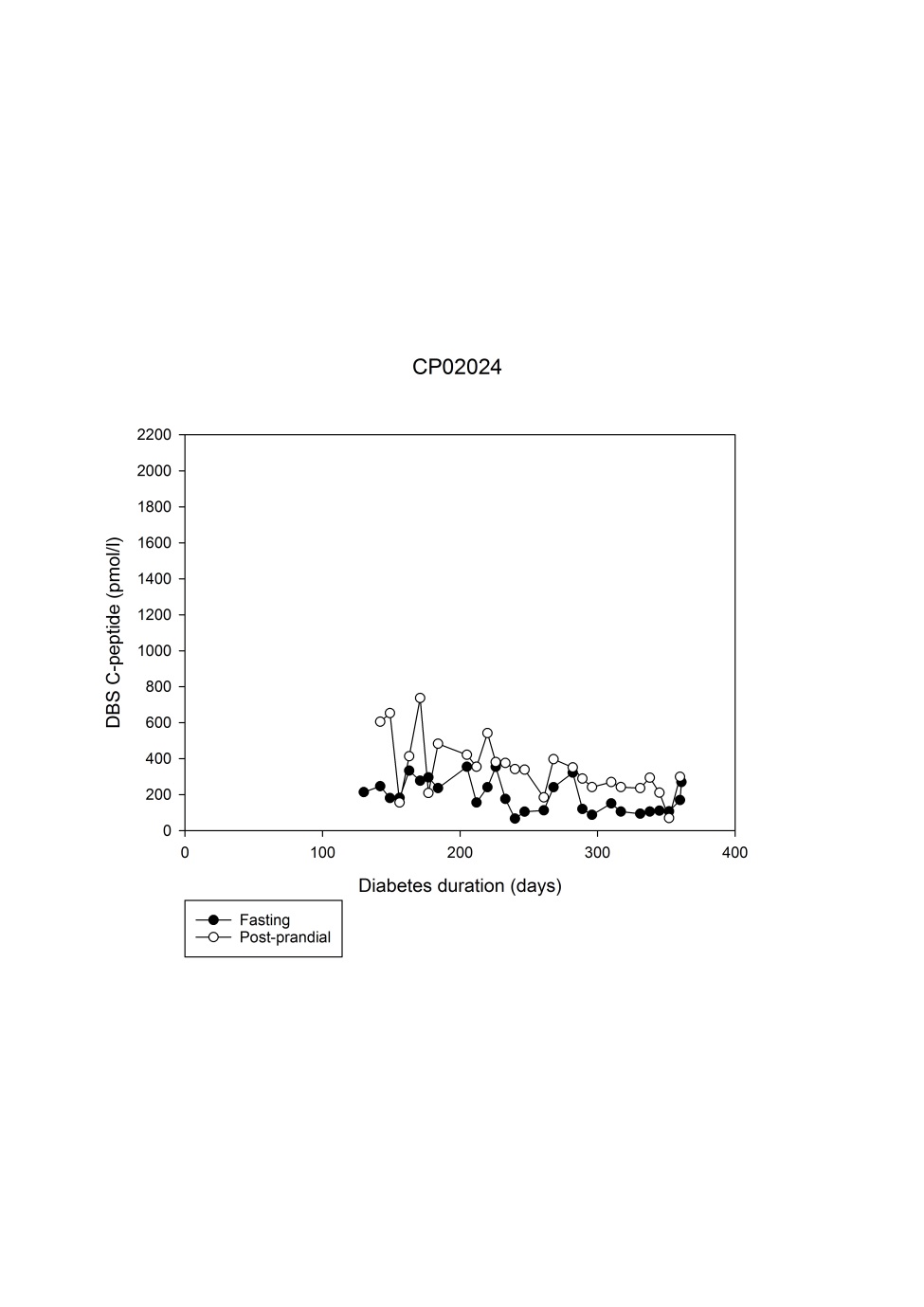

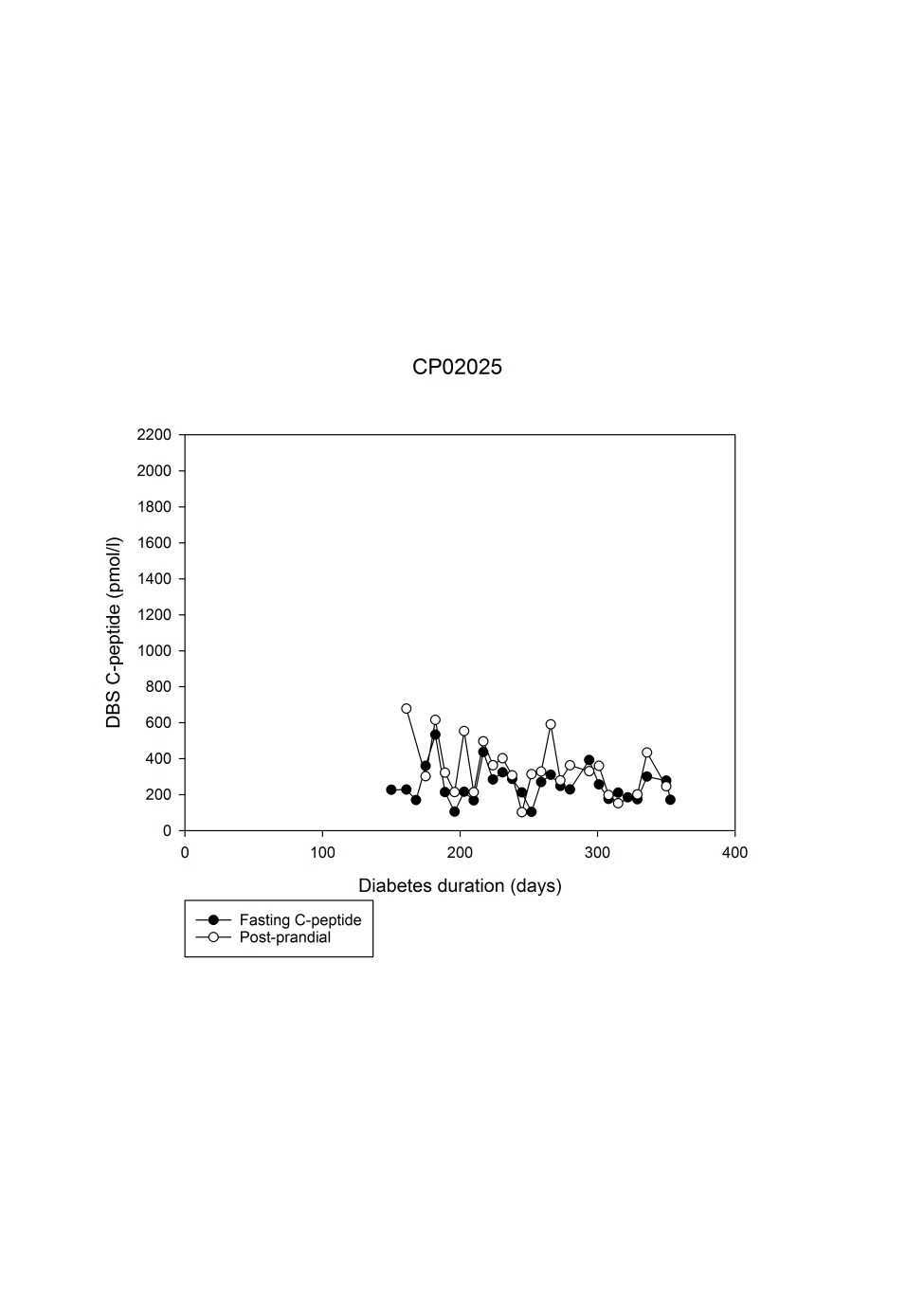

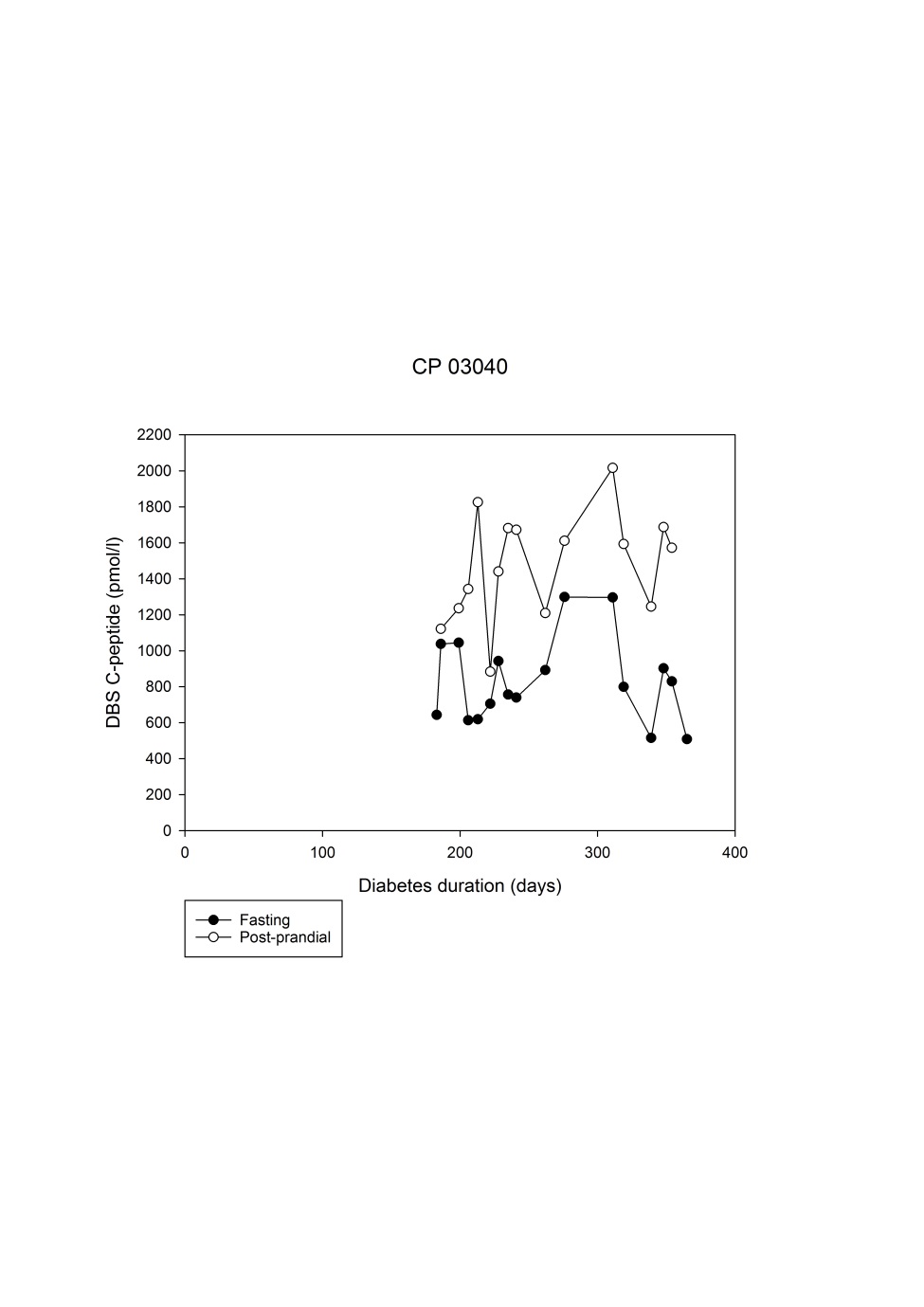

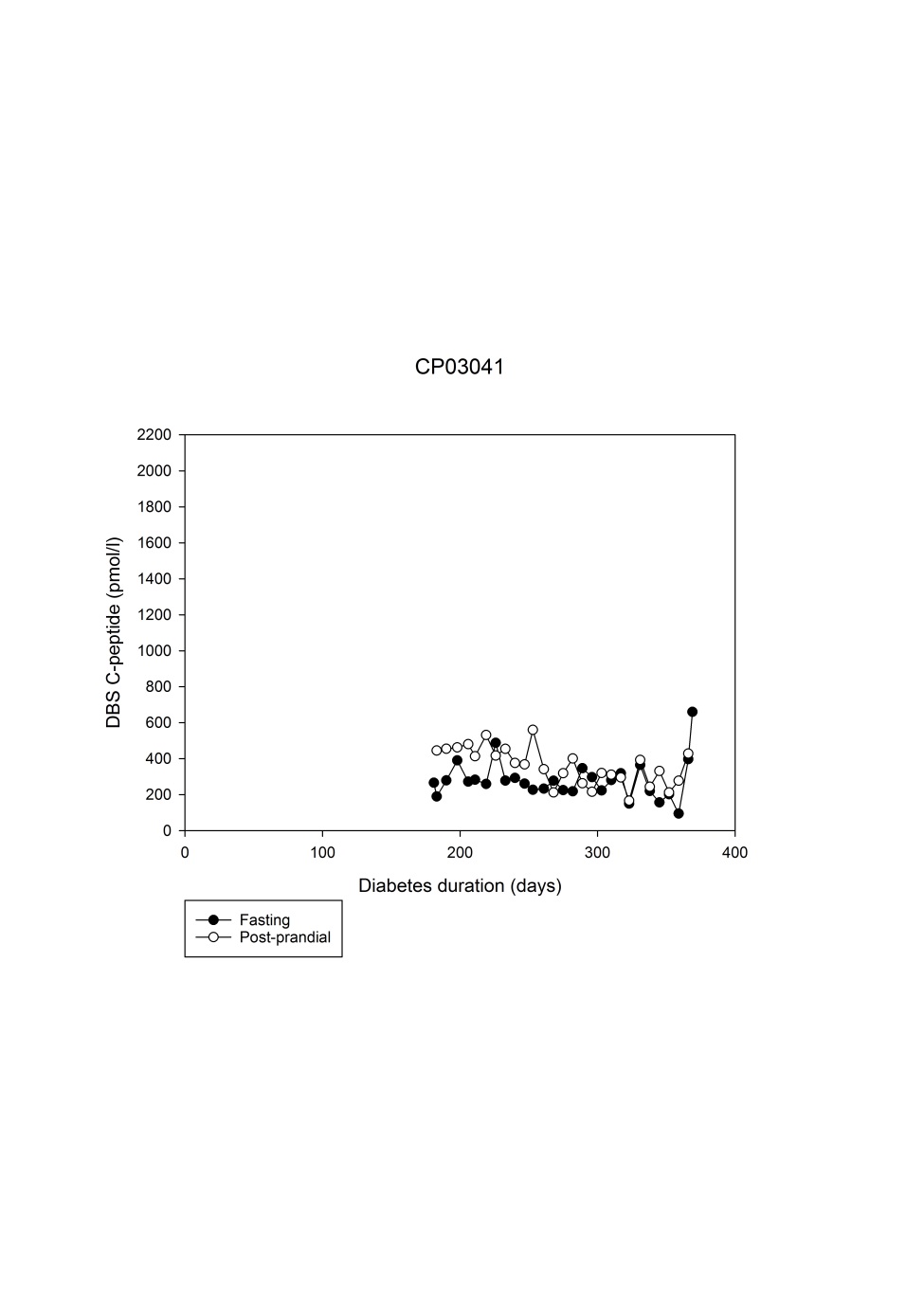

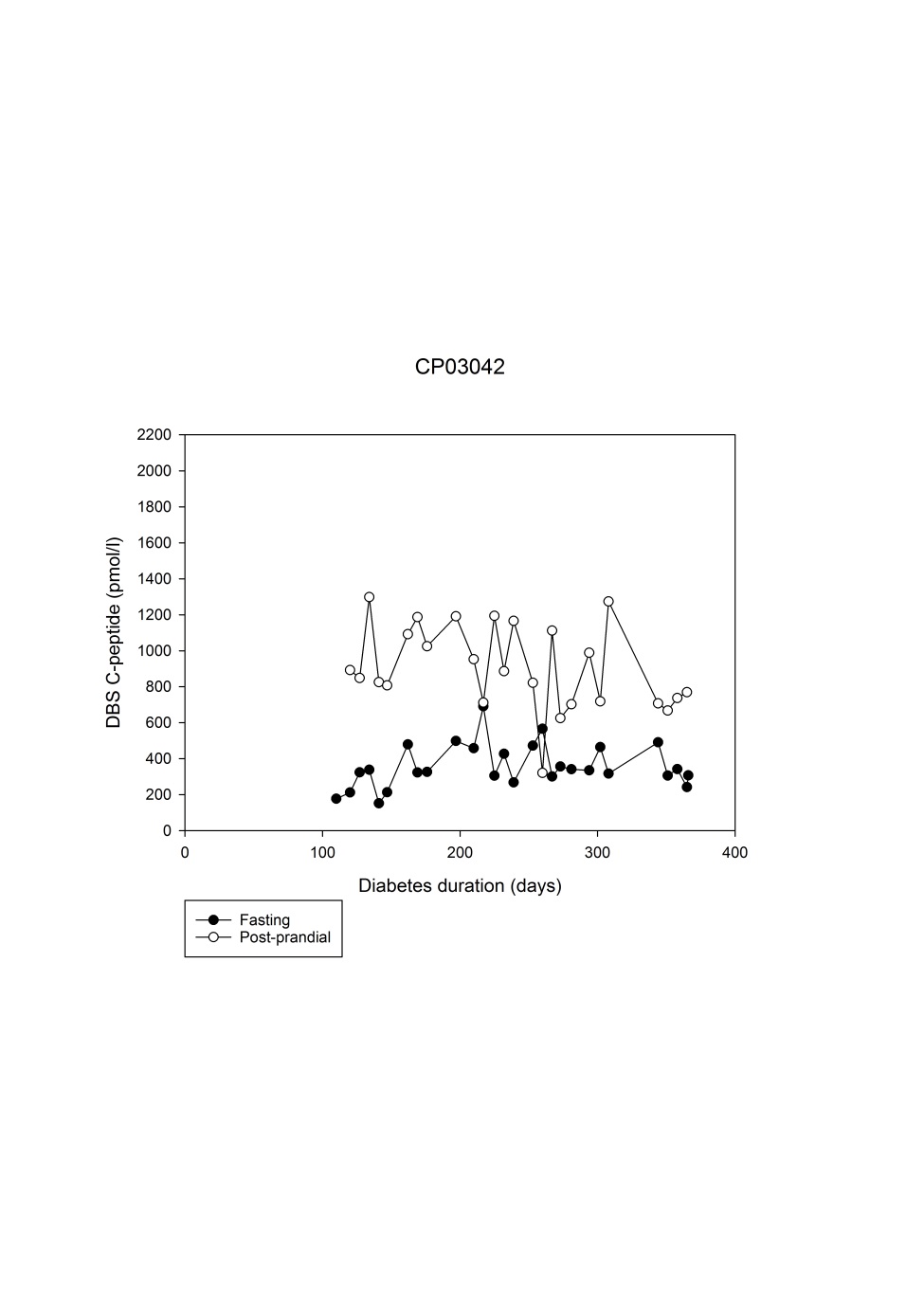

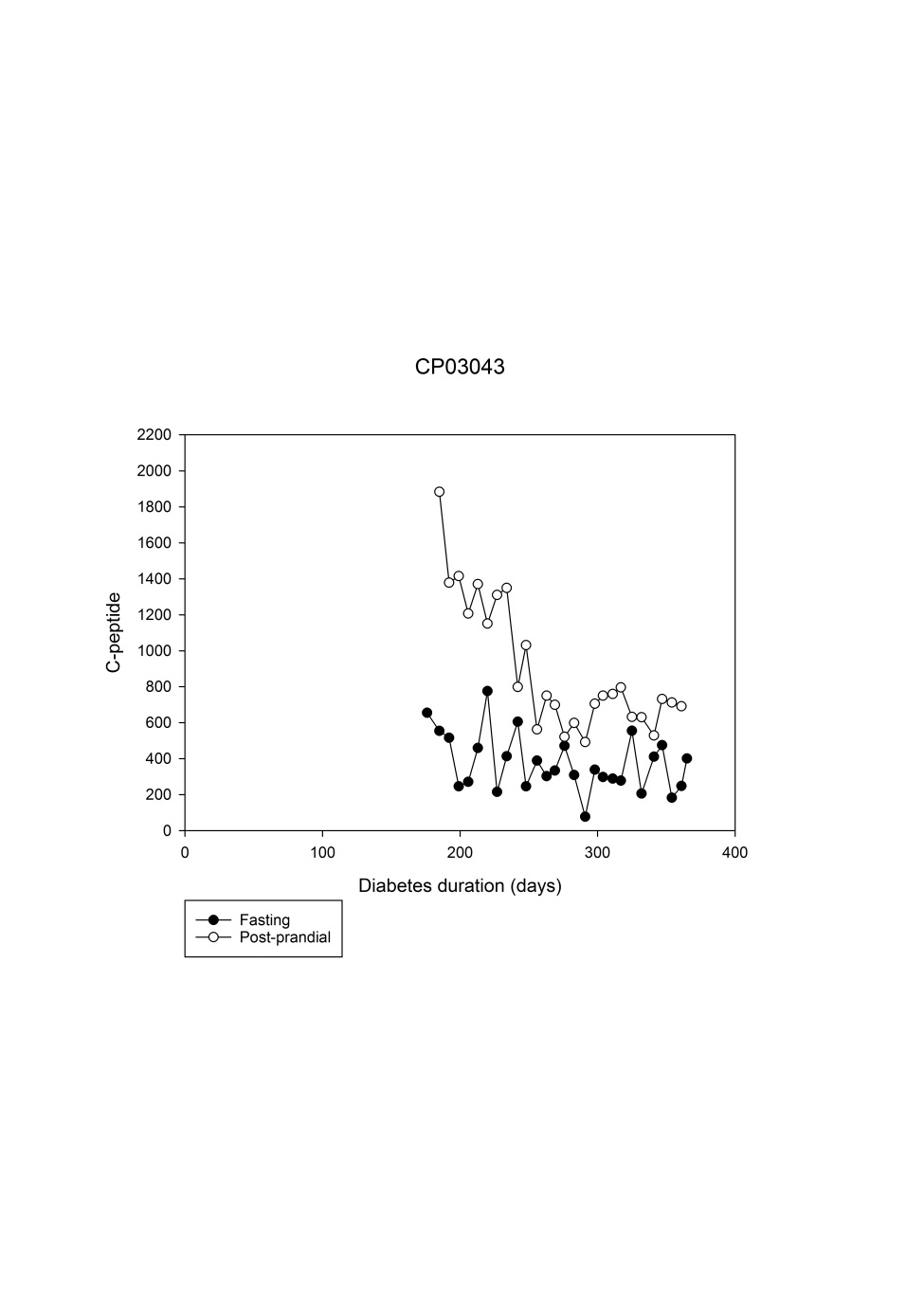

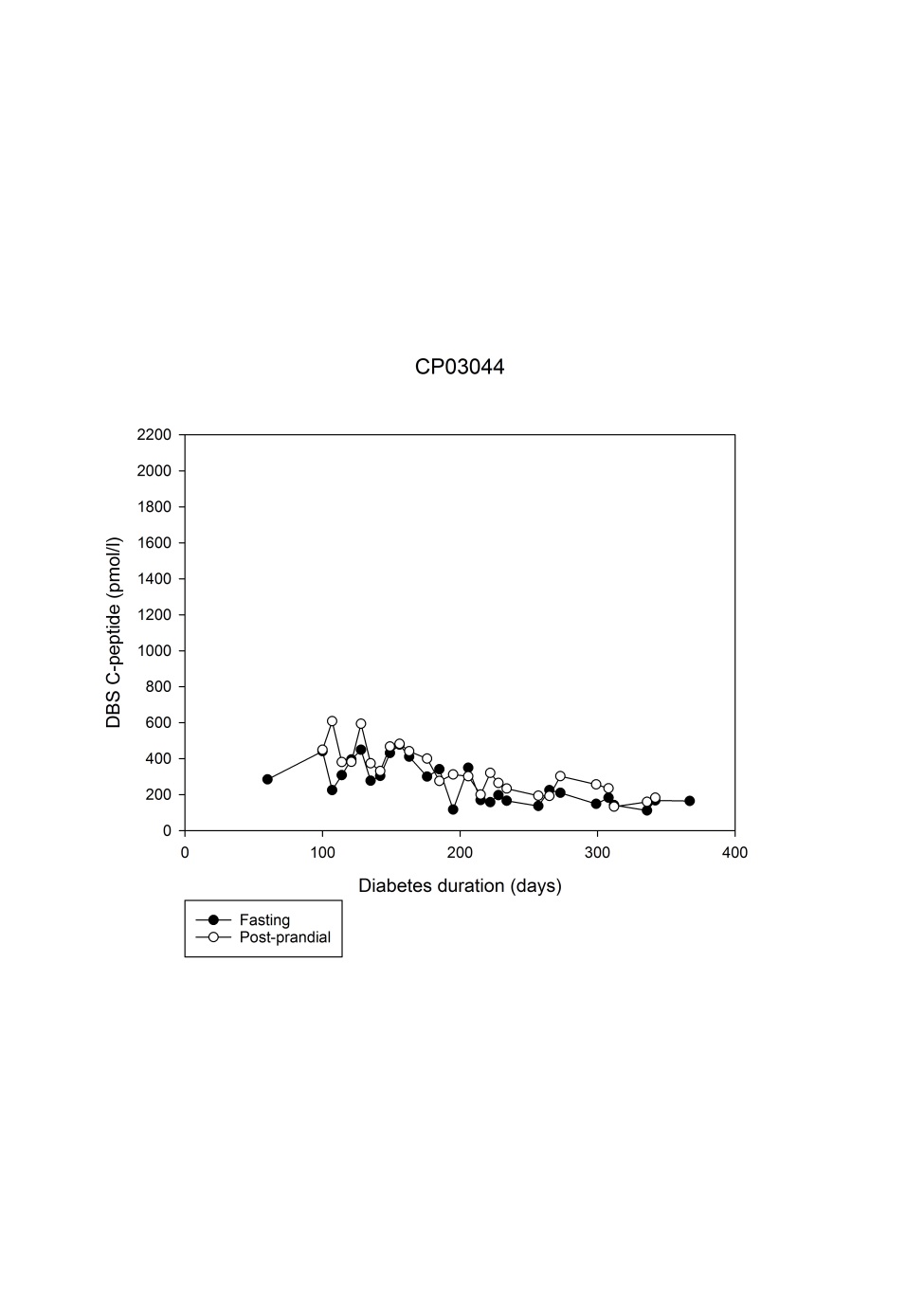

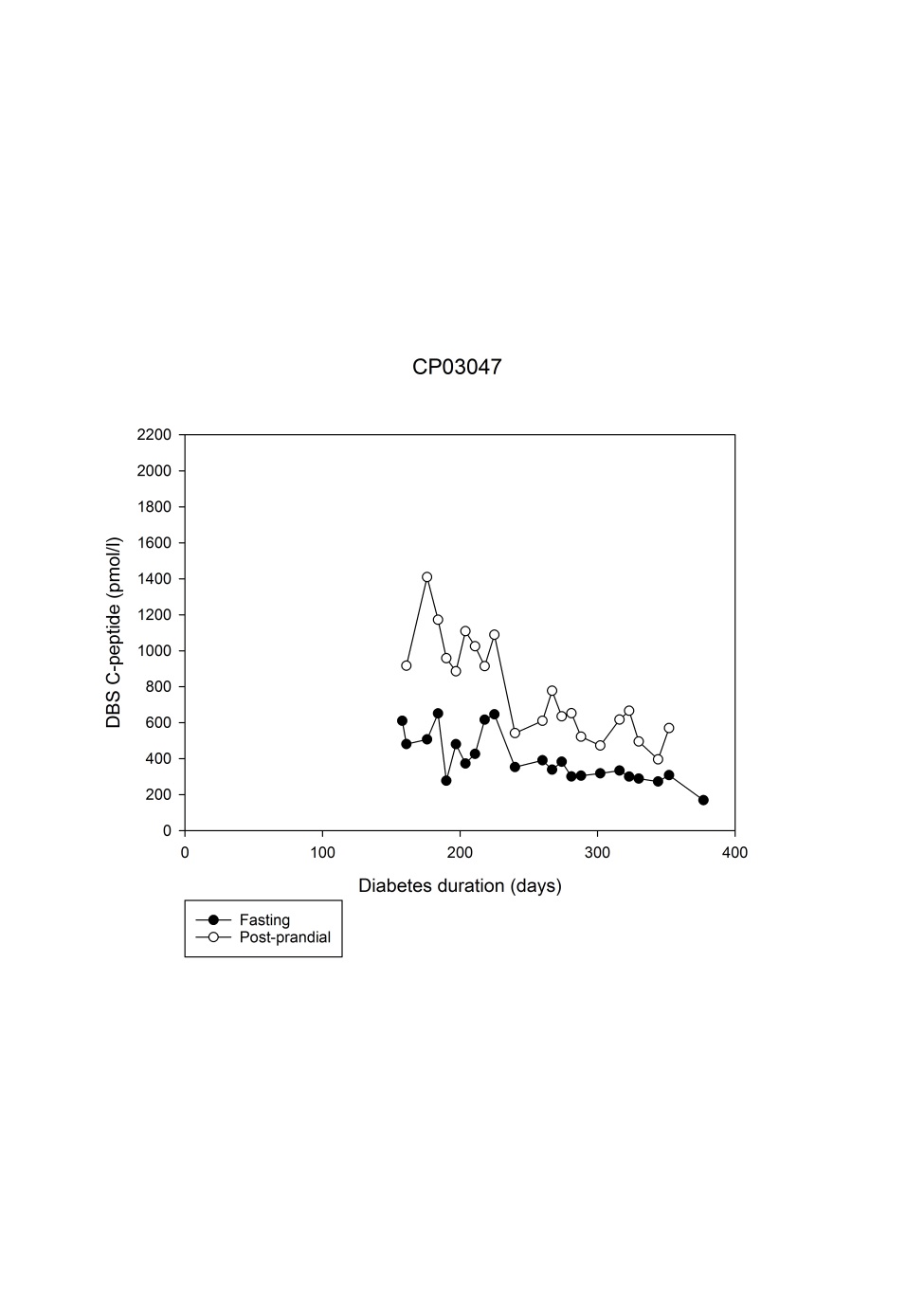

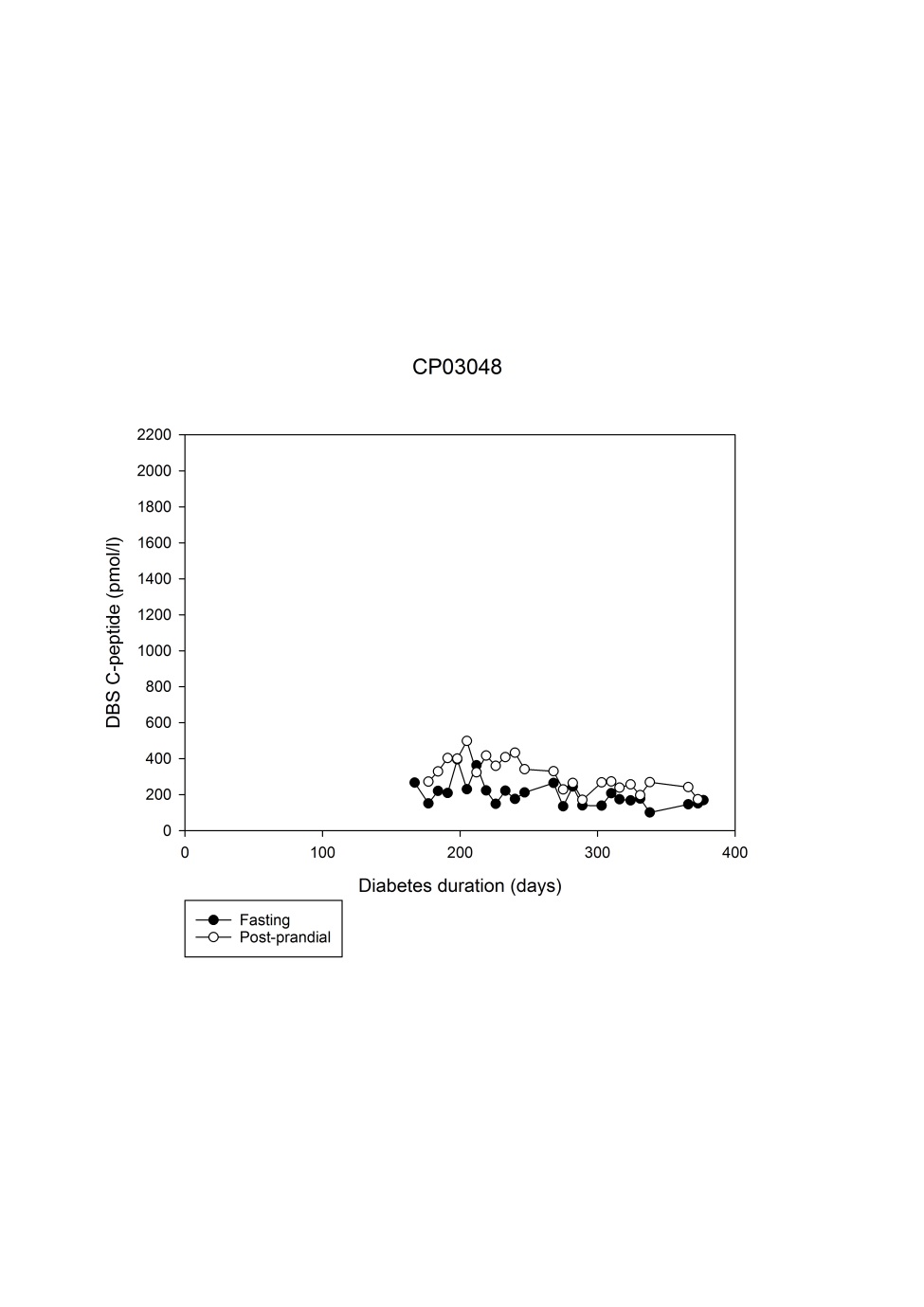

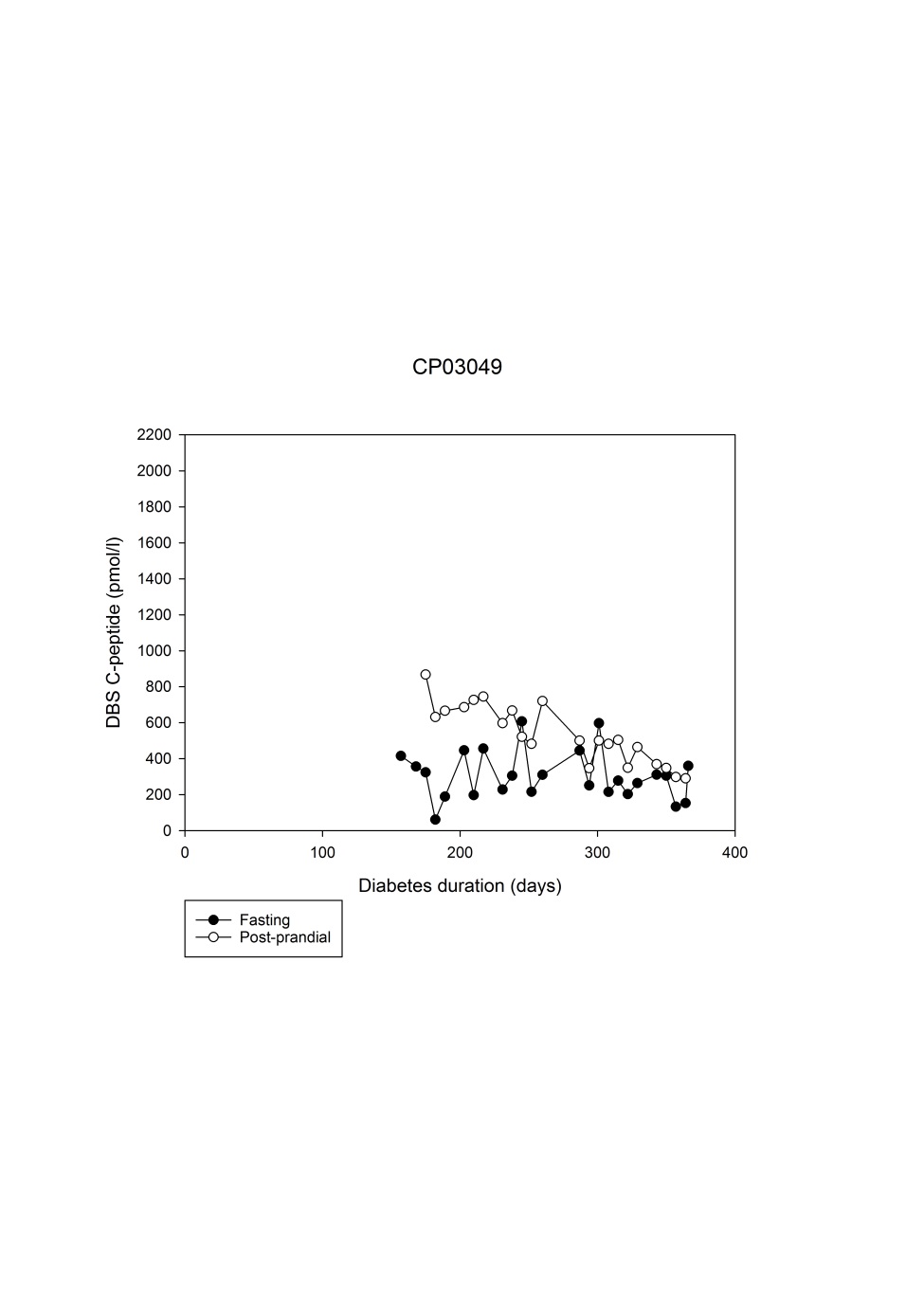

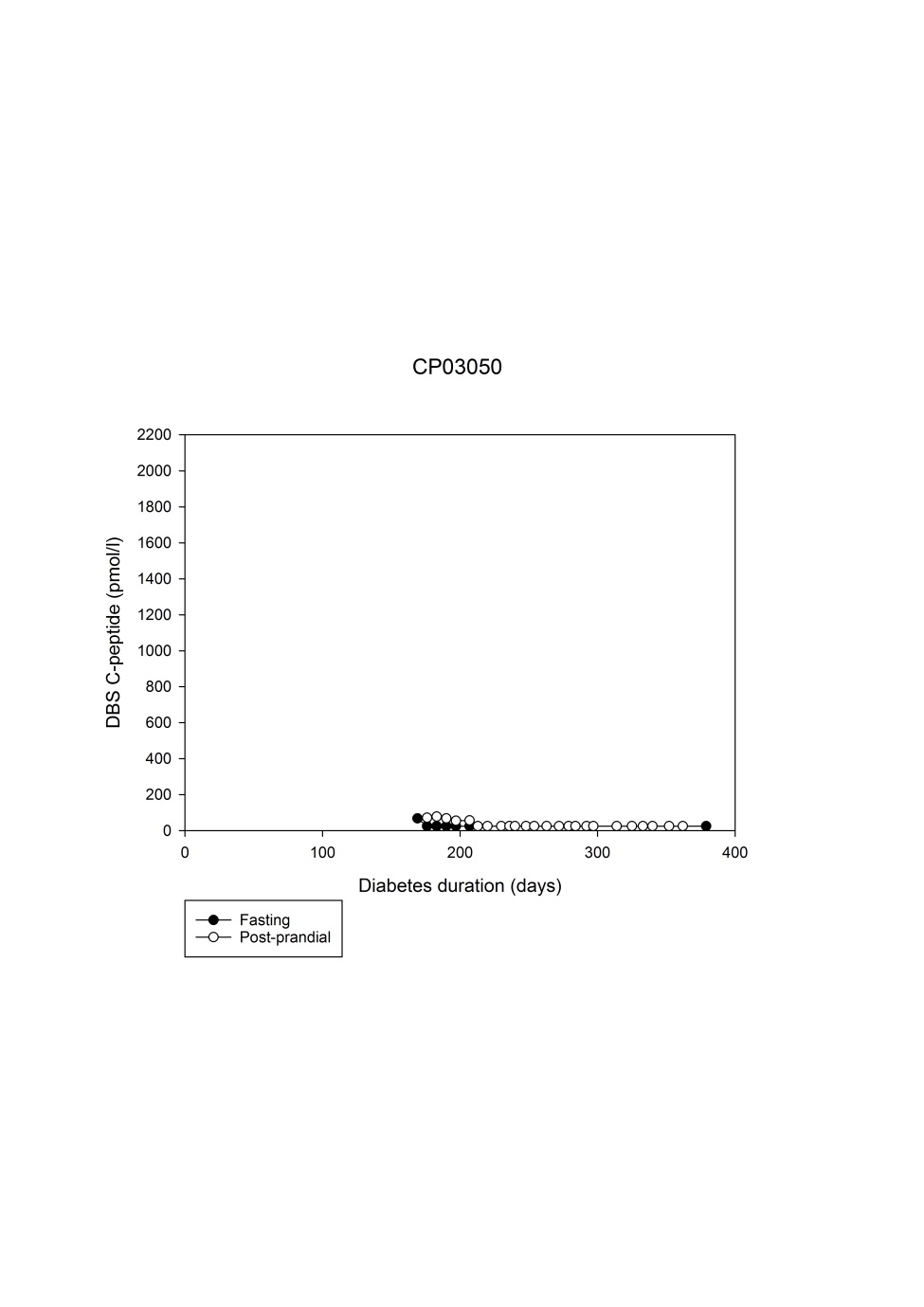

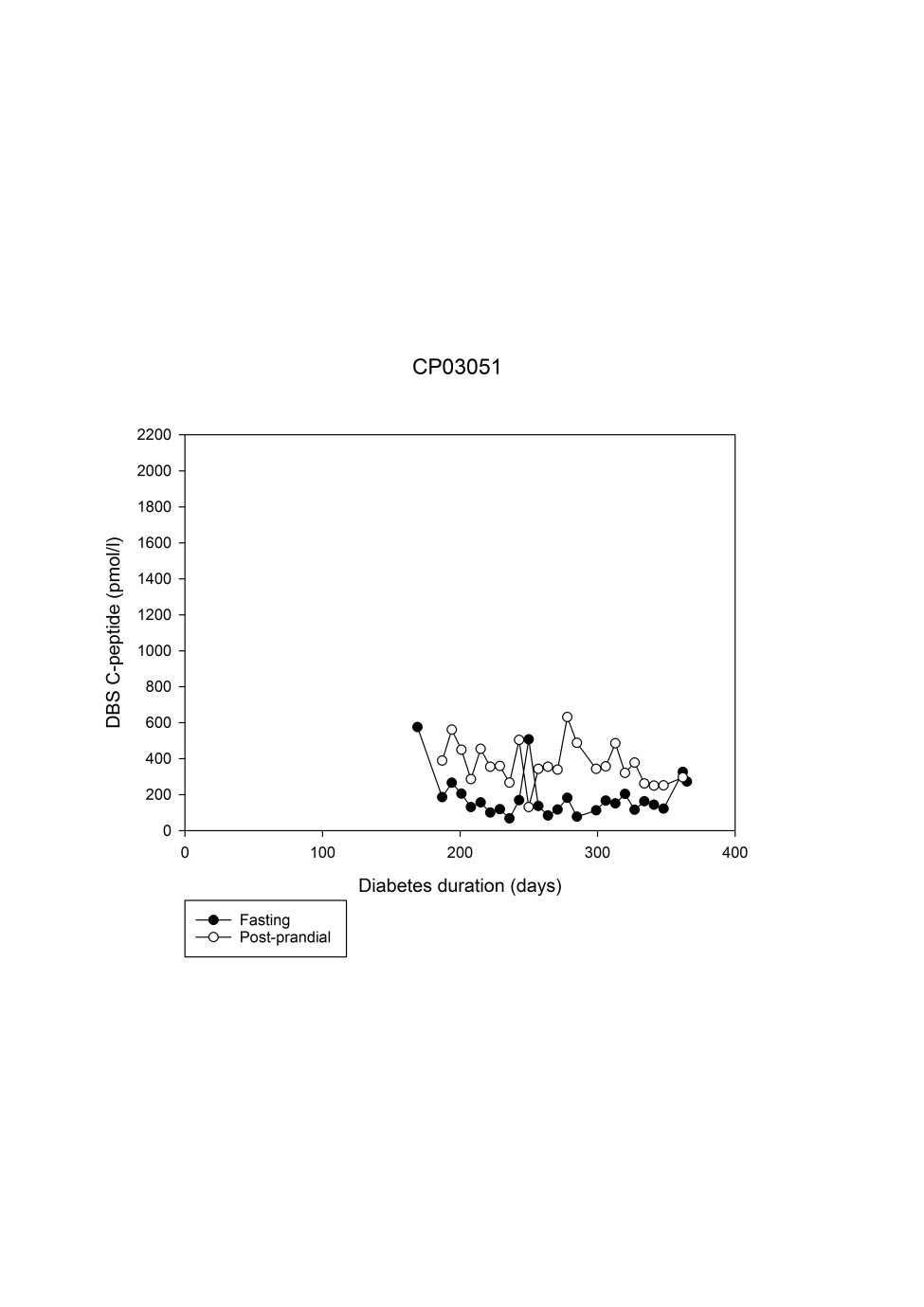

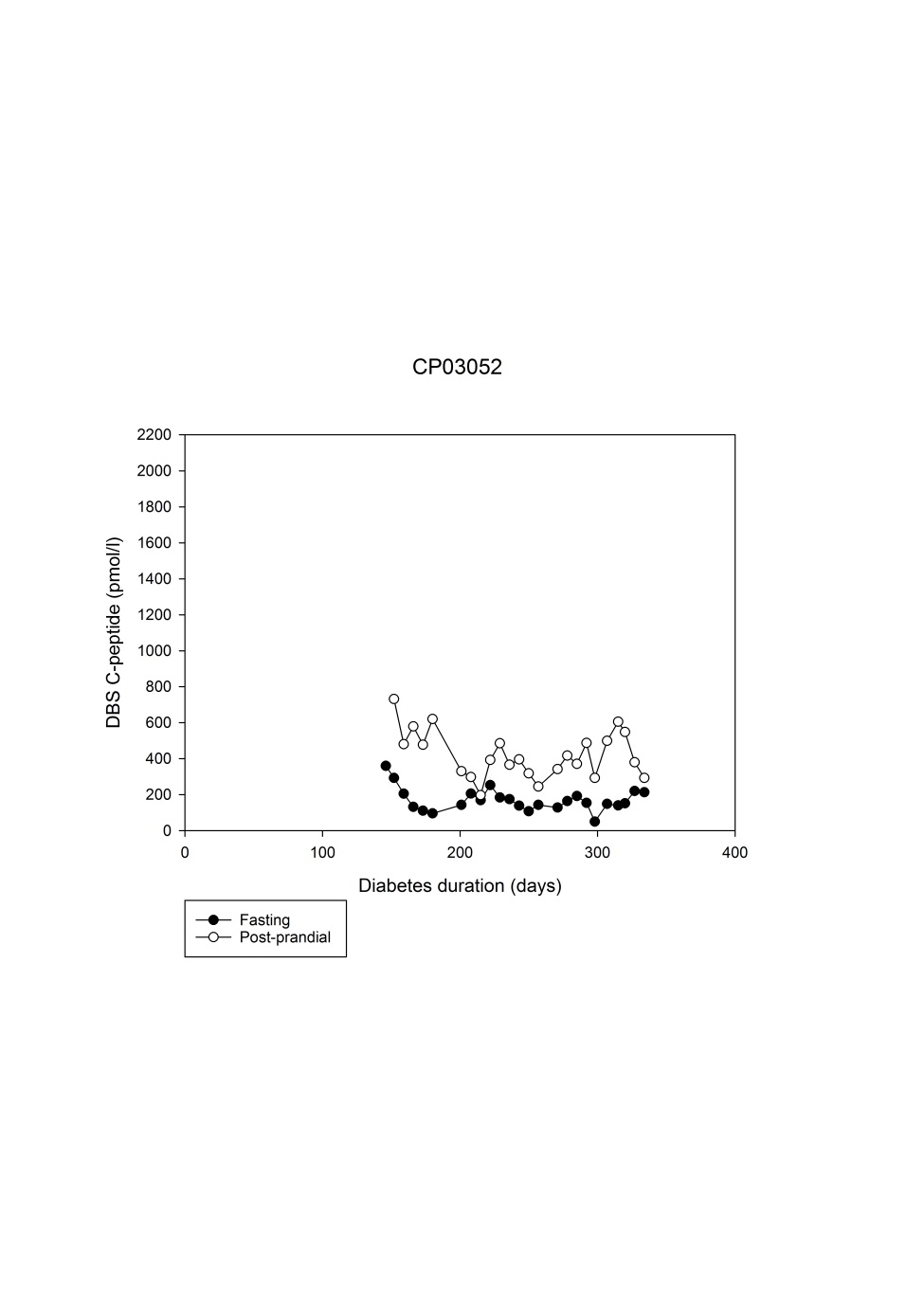


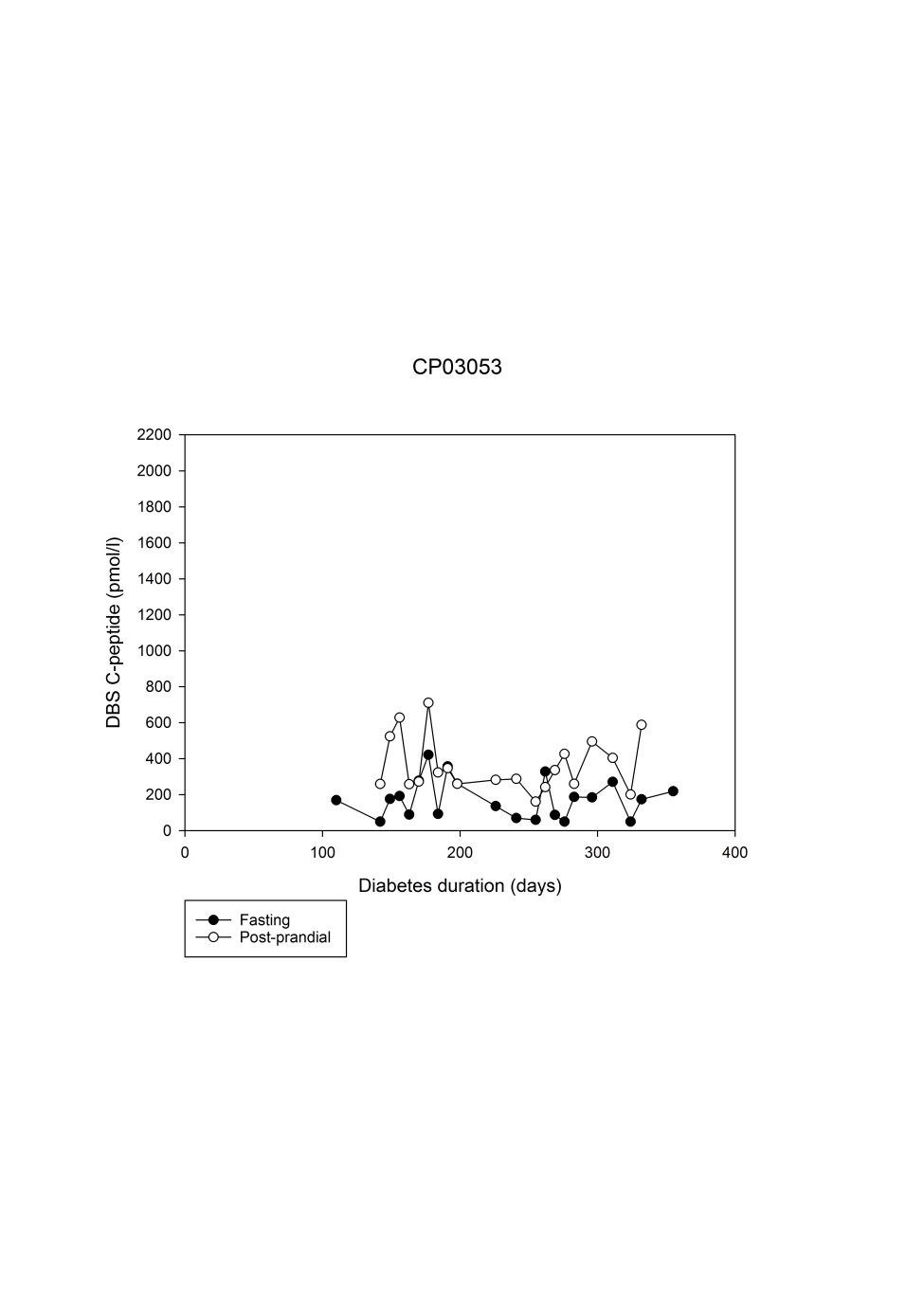

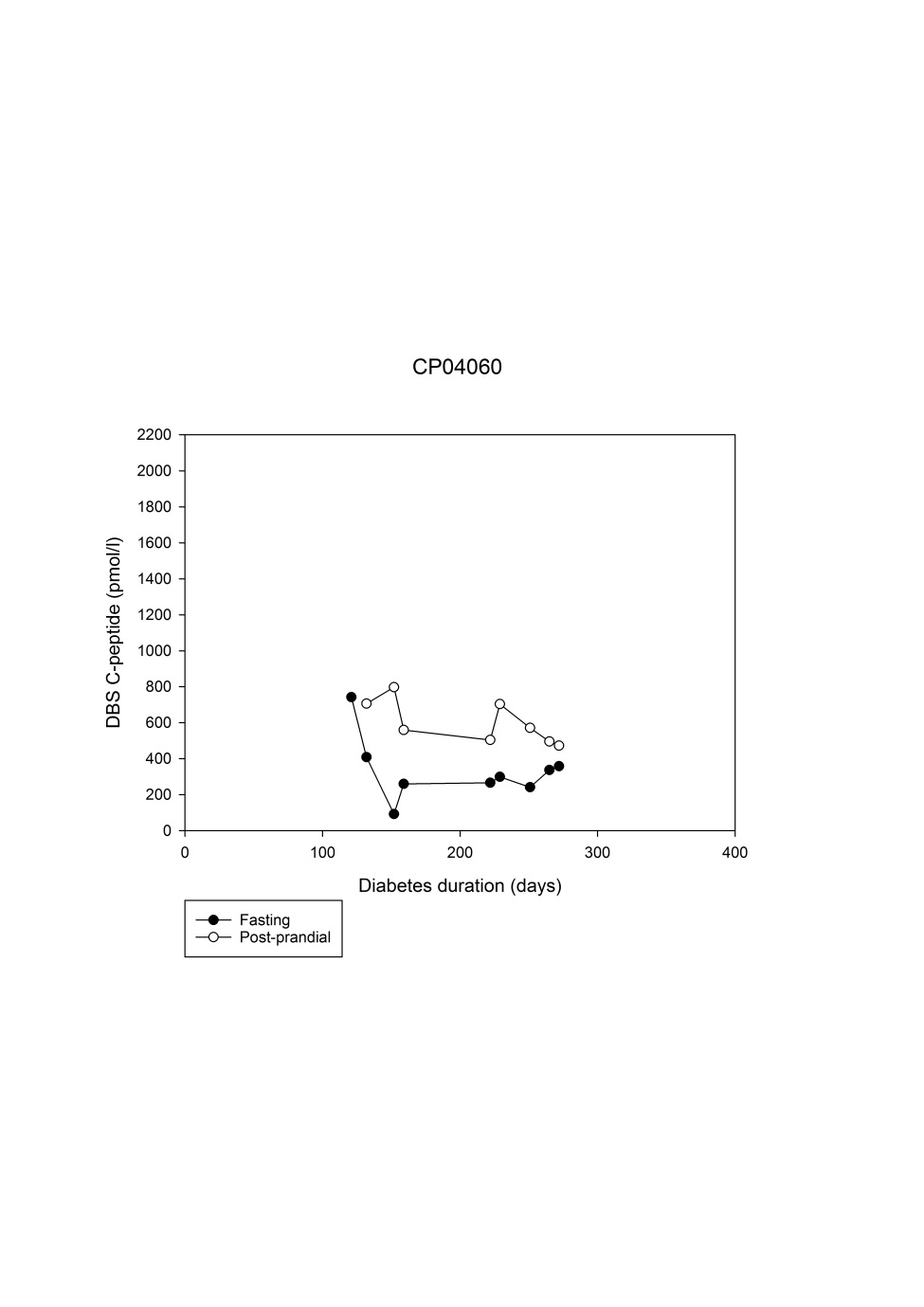

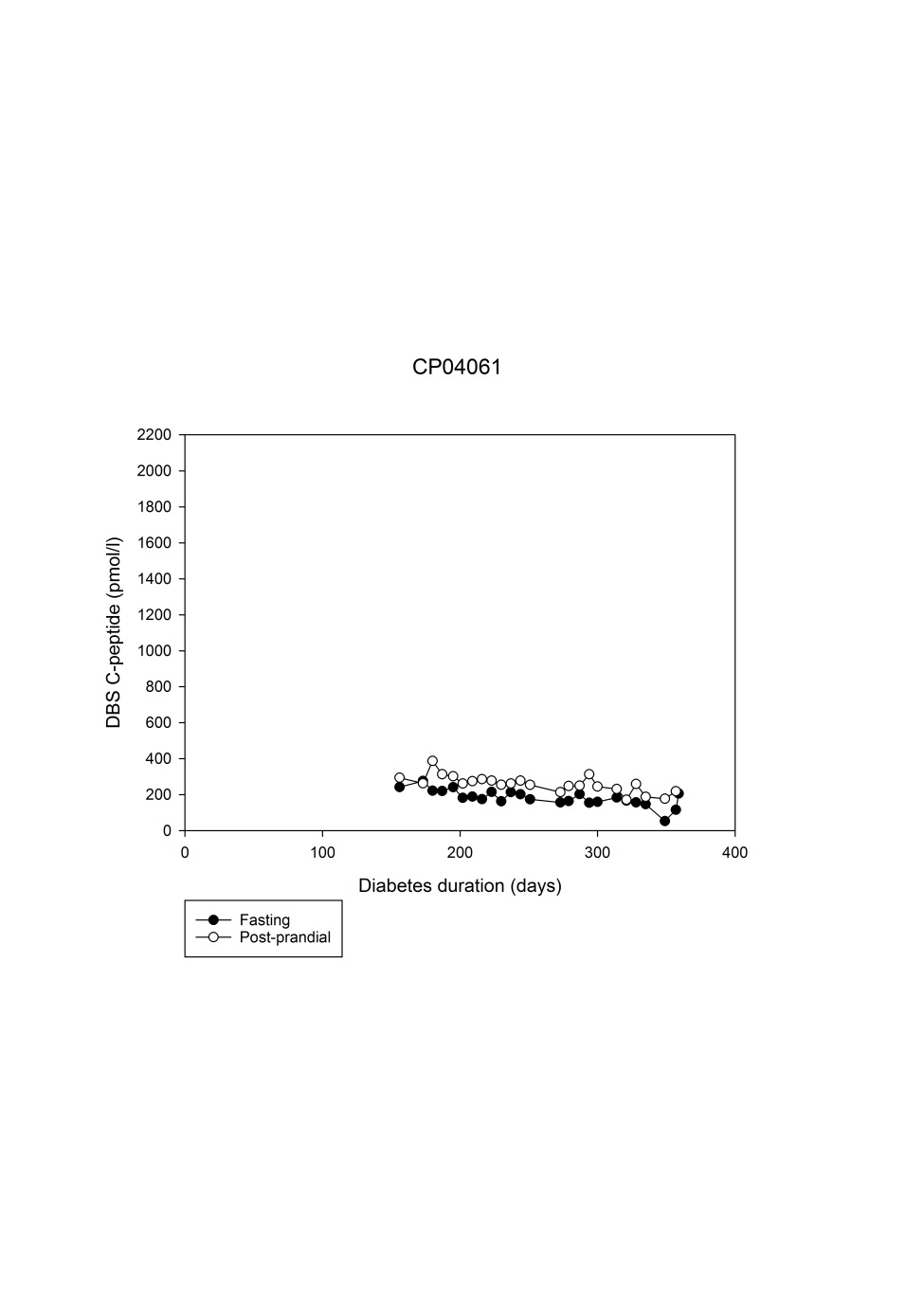

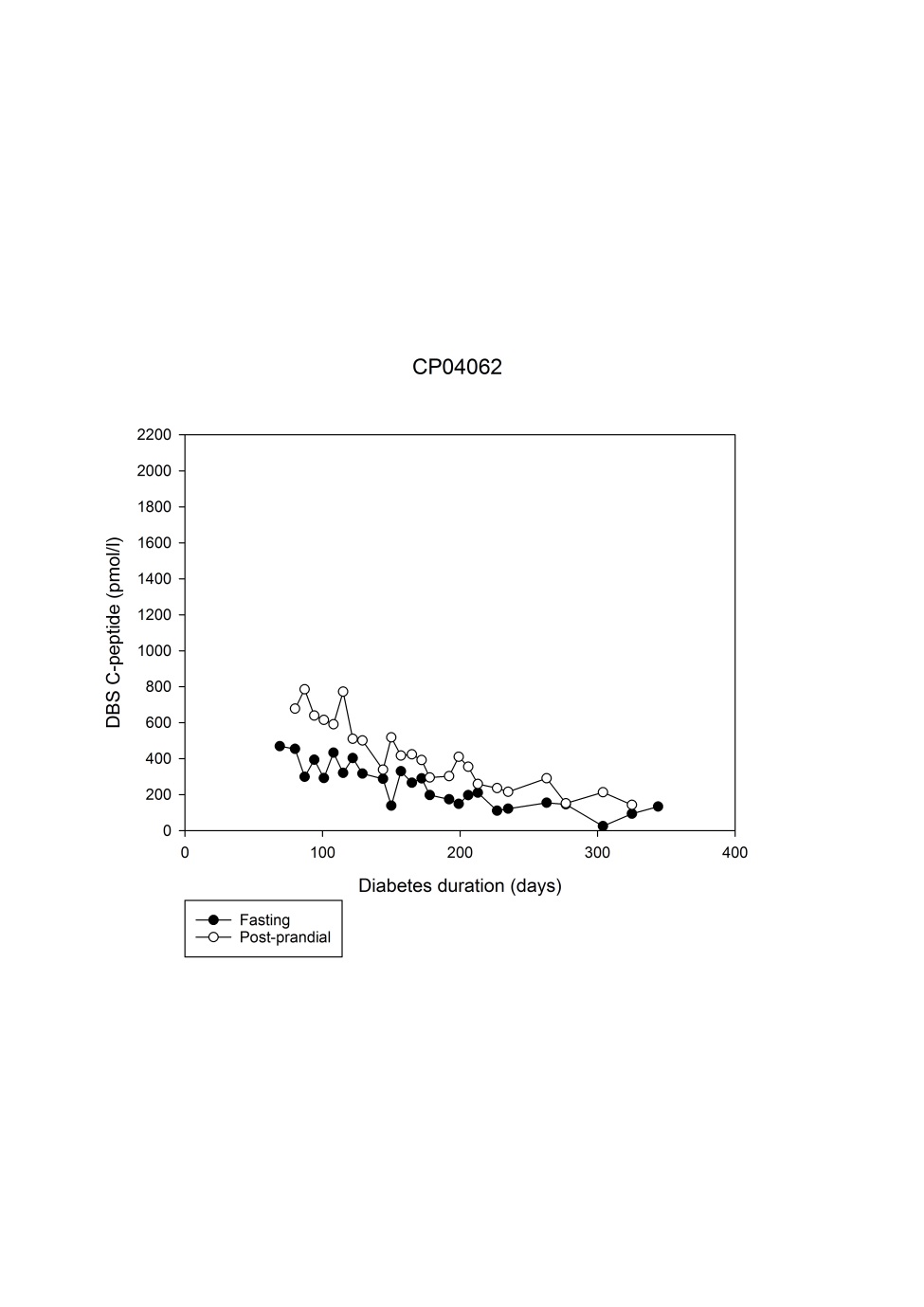


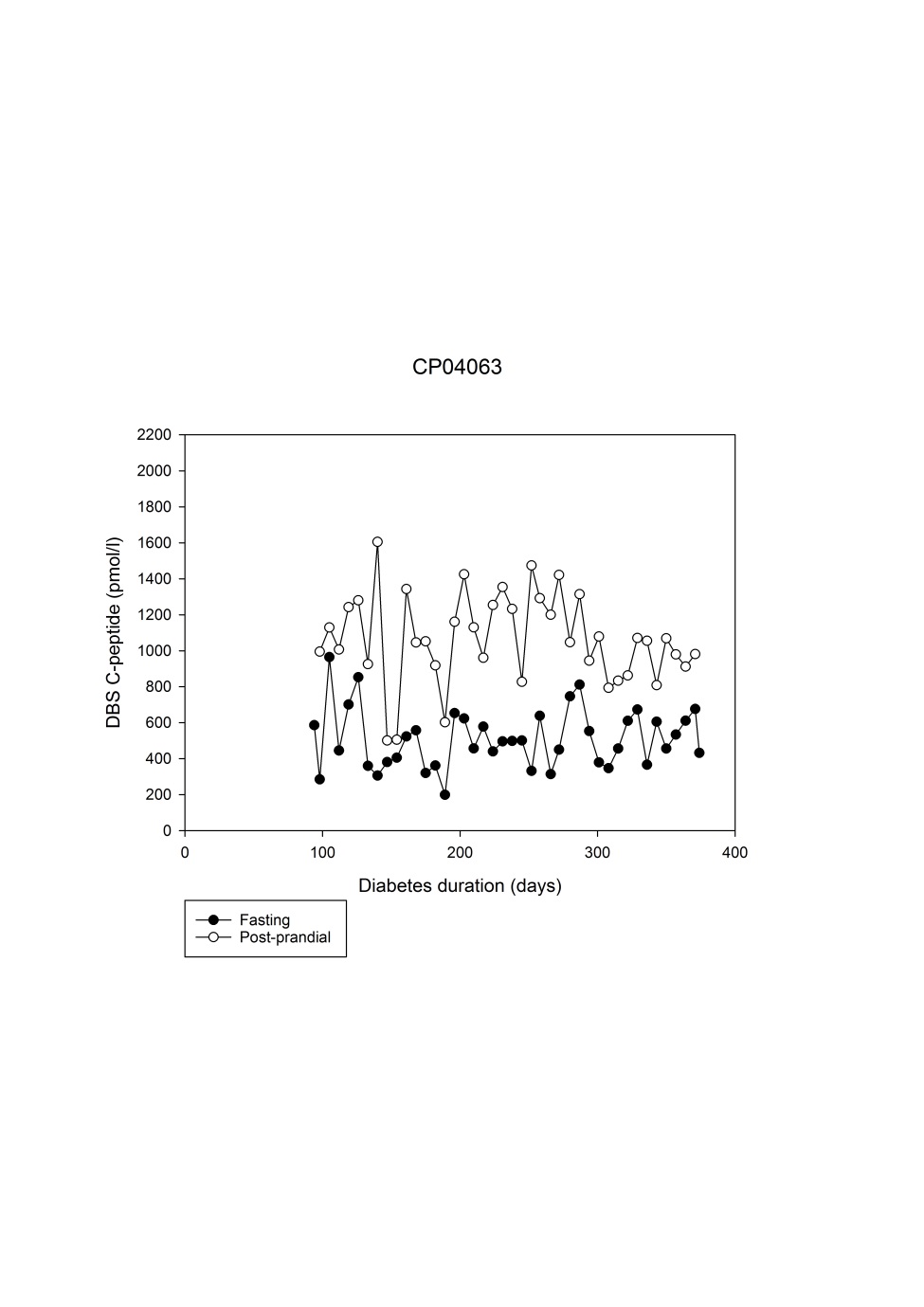

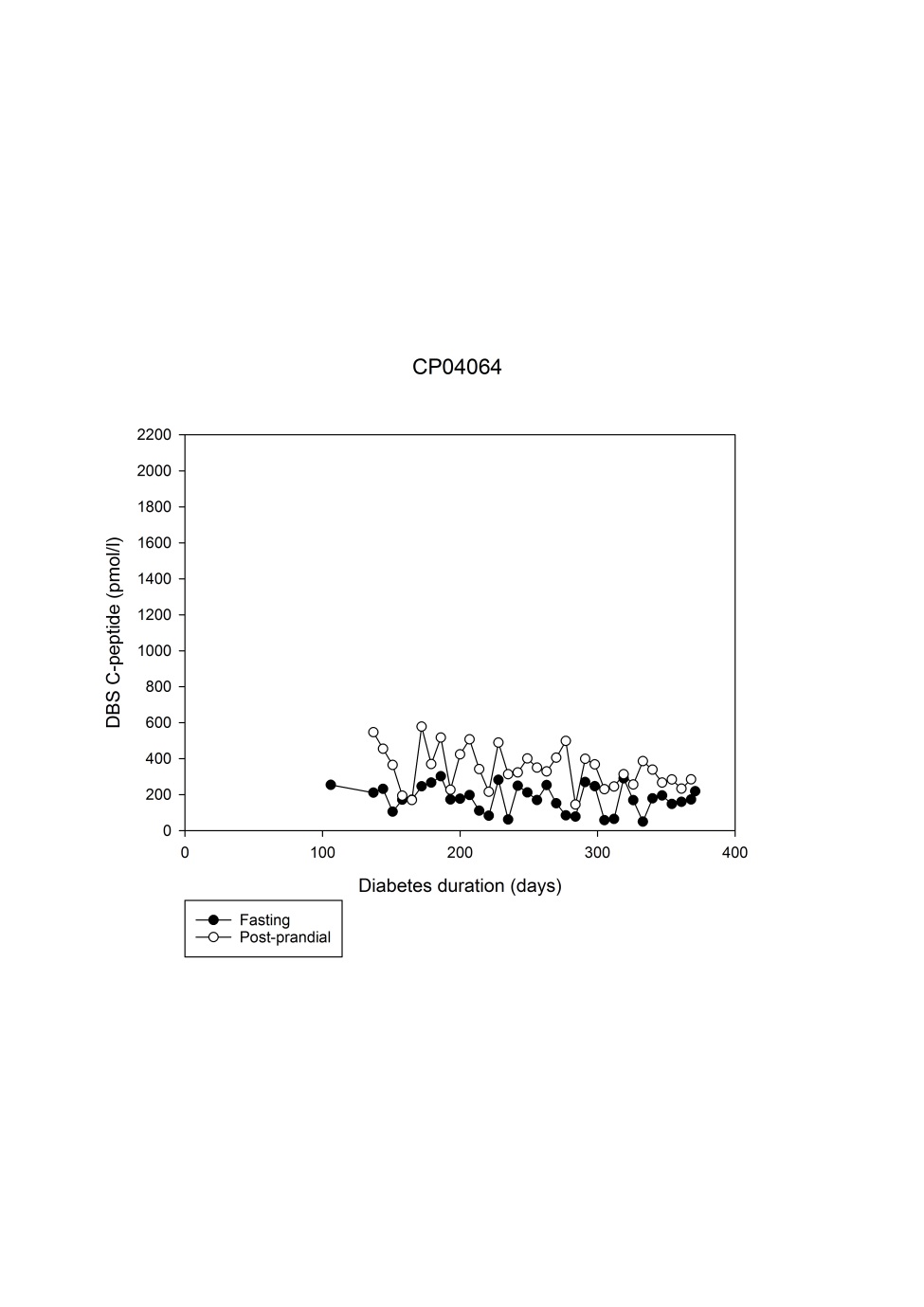


**Supplementary figure 3a.** Glucose responsiveness: Marginal effect of fasting glucose on fasting DBS C-peptide levels versus diabetes duration.

**
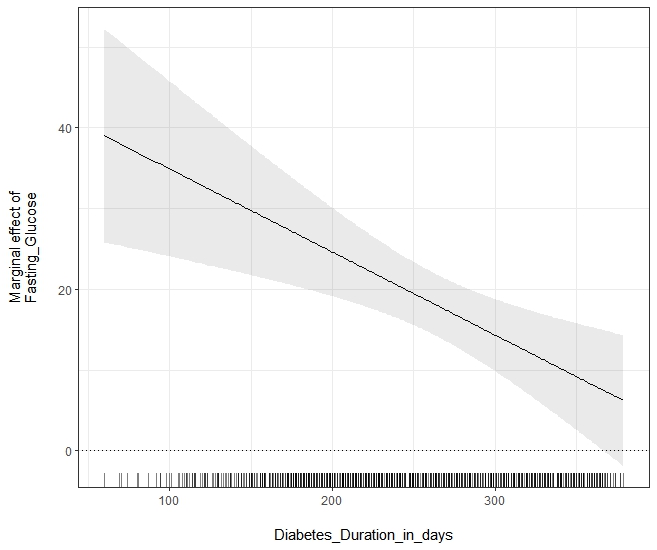
**

**Supplementary figure 3b.** Glucose responsiveness: Marginal effect of post-prandial glucose on post-prandial DBS C-peptide levels versus diabetes duration.

**
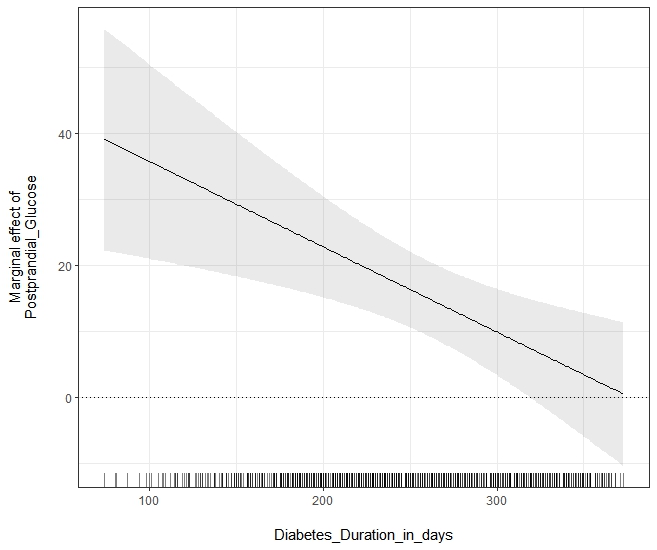
**

**Supplementary figure 3c.** Glucose responsiveness: Marginal effect of delta glucose on DBS C-peptide increment versus diabetes duration.


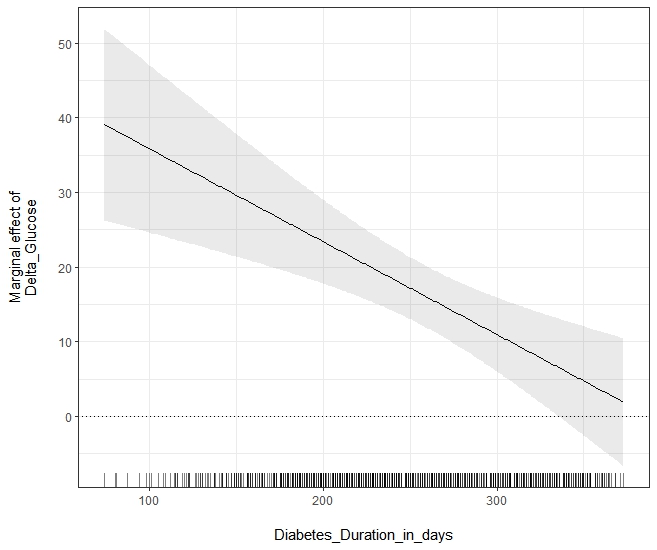

Supplement: Supplemental Figures [file jc.2018-00500.sf1.docx]
